# Translation accuracy in *E. coli*

**DOI:** 10.1093/nar/gkag674

**Published:** 2026-07-06

**Authors:** Ryan Stikeleather, Farhan Ali, Wei-Chin Ho, Tim Licknack, Michael Lynch

**Affiliations:** Biodesign Center for Mechanisms of Evolution, Arizona State University, S McAllister Ave., Tempe, AZ 85281, United States; Biodesign Center for Mechanisms of Evolution, Arizona State University, S McAllister Ave., Tempe, AZ 85281, United States; Biodesign Center for Mechanisms of Evolution, Arizona State University, S McAllister Ave., Tempe, AZ 85281, United States; Department of Biology, University of Texas at Tyler, University Blvd., Tyler, TX 75799, United States; Biodesign Center for Mechanisms of Evolution, Arizona State University, S McAllister Ave., Tempe, AZ 85281, United States; Biodesign Center for Mechanisms of Evolution, Arizona State University, S McAllister Ave., Tempe, AZ 85281, United States

## Abstract

Translation is a fundamental process of life, yet methods to systematically investigate its fidelity have been limited. Most previous estimates of translation-error rates have relied on reporter assays that evaluate only a single codon and fail to capture the full spectrum of translation errors. Here, we present a proteome-wide analysis of mass spectrometry data that directly estimates nearly all pairwise amino-acid substitution rates, revealing mistranslation rates and spectra per amino acid and per codon. Applying this method to ribosomal variants of *Escherichia coli* reported to differ in translation fidelity, we found no significant differences among their overall error rates, estimated here at 2 per 1000 amino acids. Instead, each variant exhibited unique mistranslation profiles; the putative error-prone variant preferentially misread near-cognate codons at the third position, with a bias that likely led to prior overestimates of its error rate. We also tested the translational-accuracy hypothesis of codon usage, which predicts that codons enriched in highly expressed genes are selected for translational accuracy. Contrary to that prediction, codons favored in highly expressed genes are not translated more accurately. These results underscore the necessity of proteome-wide measures of translation accuracy and highlight the limitations of single-codon approaches for characterizing translation fidelity.

## Graphical Abstract

**Graphical Abstract:**
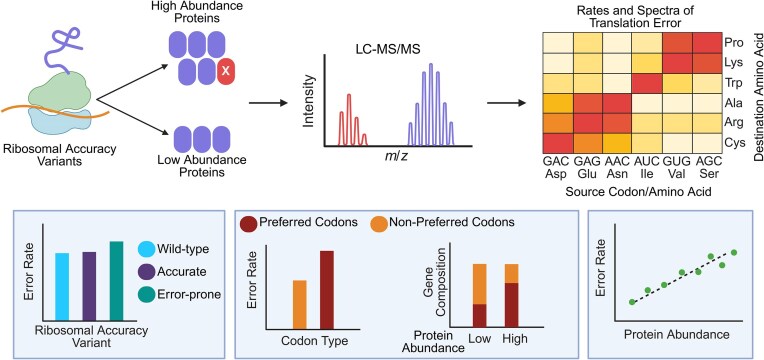

## Introduction

Translation of mRNA into protein is the most error-prone step of gene expression, yet proteins are essential for cellular function and fitness [1]. The total translation-error rate of an organism is the composite of the rates associated with ribosomal misreading and transfer RNA (tRNA) mischarging. Estimates of fidelities for both misreading and mischarging are similar, on the order of 10^−4^–10^−3^ per codon, which is at least an order of magnitude higher than the transcript-error rate [2–5]. In some cases, mischarging rates have even been reported to be on the order of 10^−2^ [6, 7]. The translation-accuracy hypothesis of codon usage [8] posits that natural selection acts on synonymous codon usage to minimize missense translation errors at conserved sites, which links ribosomal fidelity to genome evolution. Testing this hypothesis requires proteome-wide, codon-level error estimates, which are inaccessible from conventional methods for estimating translation-error rates that rely on single-codon, or even single-nucleotide challenges via dipeptide formation and reporter assays [3, 9]. Here, we present a mass spectrometry-based method that estimates proteome-wide error rates through a direct database search against defined substitution variants, enabling codon-level resolution across the detectable *Escherichia coli* proteome.

We apply this method to estimate the translation-error rates for three *E. coli* strains, each carrying a distinct ribosomal mutation that has been previously characterized to affect fidelity of translation [3, 10]. These *E. coli* strains are all derived from a Xac *E. coli* [*ara*, Δ*lacproAB gyrA, rpoB, argE* (amber)], otherwise considered here to be the wild-type variant, with an unmodified ribosome. The restrictive (*res*) ribosomal variant, with putatively improved accuracy, contains a K43N mutation in RpsL, an amino-acid site that contacts a decoding nucleotide C912 [3]. The putative error-prone ribosomal variant, or the ribosomal ambiguity (*ram*) strain, contains a 5-nt deletion in *rpsD* from nucleotides 528–532, resulting in a 180-amino-acid truncation of the S4 protein that usually interacts with S5 [3].

The phenotypic characterizations of these strains were originally done using both dipeptide formation and dual-reporter luciferase assays [3, 10, 11]. Reporter assays capture more factors important to translation, but they are still limited to the evaluation of one codon of one protein. In the case of the luciferase dual-reporter system, evaluation of the translation error is dependent on the misreading of an Asn AAU codon under the presumption that a misread would most likely incorporate a Lys (AAA). However, in firefly luciferase, 21 amino-acid sites affect the luminescence of the protein either positively or negatively depending on the substitution [12]. Errors that do not improve luminescence will go undetected. The proteome-wide error estimates presented here allow us to re-evaluate these fidelity phenotypes.

In addition to these technical limitations, reporter assays are also restricted to species that can be genetically manipulated. Our approach should be widely applicable for determining translation-error rates and the spectrum of amino-acid and codon substitution rates, as well as protein- and codon-level error rates. The proteome-wide error estimates obtained here enable a direct test of the translation-accuracy hypothesis, as well as a related hypothesis, that highly abundant proteins should exhibit lower translation error rates [13].

## Materials and methods

### Culture conditions

*Escherichia coli* ribosomal variants (a generous gift from Dr Hani Zaher) were grown overnight at 37°C in Luria–Bertani broth (Miller formulation) with aeration. Cultures were grown to stationary phase prior to protein purification.

### Protein purification and peptide preparation

Cells were pelleted via centrifugation and frozen prior to lysis. Lysis was achieved by incubating the pellets in 25 µl pH 7.5 lysis buffer (pH adjusted with phosphoric acid) consisting of 5% sodium dodecyl sulfate and 50 mM triethylammonium bicarbonate (TEAB) at 95°C for 10 min. Samples were kept on ice unless otherwise stated. Samples were then clarified by centrifugation for 10 min (13 000 RCF). Proteins (the supernatant) were reduced via the addition of dithiothreitol (DTT) to a concentration of 50 mM with a 10-min incubation at 95°C. Iodoacetamide (IAA) was added to a concentration of 40 mM, and samples were incubated at room temperature, in the dark, for 30 min to irreversibly modify cysteines to carbamidomethyl cysteine, preventing them from forming disulfide bonds. Phosphoric acid was added to a final concentration of 5.5%, then 2 µl of 1 µg/µl trypsin was added to the acidified samples. S-trap binding buffer (100 mM TEAB in 90% methanol, pH 7.1) was then added to the peptide samples (volume equal to six times the sum total of DTT, IAA, and phosphoric acid). Samples were then transferred to S-trap columns and centrifuged at 4000 RCF for 1 min. The column was washed four times with 150 µl of the S-trap binding buffer. On-column trypsin digestion was performed by adding 25 µl of 50 mM TEAB, pH 8, containing 0.5 µg of trypsin to the top of the protein trap. The column was then incubated for 1.5 h at 47°C. Peptides were finally eluted three times (centrifugation of 4000 RCF for 1 min), first with 40 µl of 50 mM, pH 8, TEAB, then with 0.2% aqueous formic acid, and then with 35 µl of 50% acetonitrile containing 0.2% formic acid. Peptides were dried in a speed vacuum for 2 h, and then resuspended in 20–40 µl of 0.1% formic acid.

### Mass spectrometer settings

Approximately 500 ng of peptides were injected onto an Ultimate 3000 HPLC (Thermo Fisher Scientific) at 0.300 nl/min and separated on a 50-cm EASY SPRAY C18 column (Thermo Fisher Scientific) before being analyzed in an Orbitrap Fusion Lumos Tribrid Mass Spectrometer (Thermo Fisher Scientific). Buffer A consisted of 99.9% LC-MS-grade water, Buffer B consisted of 99.9% LC-MS-grade acetonitrile, and both buffers contained 0.1% formic acid. A 5-min equilibration preceded a 60-min analytical gradient of 1%–35% B and a 5-min column wash at 80% B. The mass spectrometer was run in database-dependent acquisition (DDA) mode with both MS1 and MS2 being detected on the Orbitrap. The MS1 full scan resolution was set to 120K, a scan range of 375–1500 *m*/*z*, charge states were set to the range of +2 to +7, the AGC target was set to 400K, and maximum injection time was 50 ms. Dynamic exclusion was set to a duration of 60 s. Precursor ions were activated by HCD with 30% collision energy, MS2 resolution was set to 30K, the AGC target was set to 50K, and the maximum injection time was 54 ms. Raw data files were analyzed in Proteome Discoverer 3.1 (Thermo Fisher Scientific).

### Whole-genome sequencing and generation of annotation files

The ribosomal variants of *E. coli* were subjected to whole-genome sequencing, specifically hybrid sequencing (a combination of Illumina and Nanopore sequencing), which was carried out by SeqCoast Genomics. DNA was extracted using a MagMax Microbiome Ultra Nucleic Acid Isolation Kit. For Illumina sequencing, samples were prepared for using the Illumina DNA Prep tagmentation kit and IDT For Illumina Unique Dual Indexes. Sequencing was performed on the Illumina NextSeq 2000 platform using a 300-cycle flow cell kit to produce 2× 150 bp paired reads. One to two percent PhiX control was spiked into the run to support optimal base calling. Read demultiplexing, read trimming, and run analytics were performed using DRAGEN v4.2.7, an on-board analysis software on the NextSeq 2000. For Nanopore sequencing, DNA samples were prepared using the Oxford Nanopore Technologies SQK-NBD114 native barcoding kit. Long Fragment Buffer was used to promote longer read lengths. Sequencing was performed on the GridION platform using a FLOW-MIN114 Spot-ON Flow Cell, R10 version with a translocation speed of 400 bp. Base calling was performed on the GridION using the super-accurate base calling model with barcode trimming enabled. Raw Illumina reads were trimmed using Trimmomatic software (v0.39), on default settings [14]. Raw Nanopore reads were trimmed using Porechop (v0.2.4) [15]. High-quality reads were assembled using the Unicycler software (v0.4.4) and annotated using BAKTA (v.1.5.1) [16, 17].

### Initial and custom database search

FASTA files for gene sequences and protein sequences were generated from genome annotation files derived from whole-genome sequencing (SeqCoast). These annotation files were processed with a custom parser that outputs FASTA files for validated protein and DNA sequences. Resultant protein FASTA files were loaded into Proteome Discoverer 3.1 as the protein database. The initial database search in Proteome Discoverer utilized the Sequest HT node for database searching, the wild-type proteome for each ribosomal variant, and the Percolator node for peptide spectral match (PSM) validation [18]. In Sequest HT, trypsin (full) was selected for proteolytic digestion with up to 2 missed cleavages allowed, a precursor mass tolerance of 10 ppm, a minimum sequence length of six amino acids, and a fragment mass tolerance of 0.02 Da. Dynamic modifications included oxidation and protein N-terminal acetylation; static modifications included only carbamidomethylation of cysteines. Default settings were used in Percolator to calculate both posterior error probabilities (PEP) and *q*-values to a target false discovery rate (FDR) of 1% [18, 19].

### Custom database generation

Protein identities were exported from Proteome Discoverer and processed through a custom python script that results in the *in silico* trypsinization of detected proteins followed by an internal homology check, then all possible amino-acid substitutions were made to peptides that passed the internal homology check, as well as length and mass filters. Length was set to six amino acids and mass was set to 500 Da; additionally, peptides containing selenocysteine were filtered out. The *in silico* trypsinization was set to allow for up to two missed cleavages due to inefficiency in the trypsin enzyme. All analysis scripts here have only been written to accommodate single substitutions. The internal homology script filters out trypsinized peptides from different proteins that differ by a single amino acid in their wild-type sequences. If two peptides were identical, then it checks the DNA sequence of both peptides, and if the DNA is also identical then the peptide is kept. In this way, the amino acid and codon level error rates are unaffected, and thereby the overall translation error rate is unaffected as well. This becomes relevant when determining protein-level error rates and protein abundance relationships, as such, those peptides are removed from analysis at that step. In sum, only proteins that were detected in the initial search were subjected to *in silico* trypsinization, an internal homology check, and mutagenesis. The resultant FASTA file contains both the wild-type parent peptides and the systematically mutagenized daughter peptides.

### Translation error rate data analysis

After performing the Proteome Discoverer search with the custom database, identified peptides were processed through a custom python pipeline. Peptides were filtered on Percolator-derived [18] PEP values for PSM validation (PEP = 0.01). Peptide sequences containing Ile to Leu or Leu to Ile substitutions were reverted to wild type, as Leu and Ile are isobaric and indistinguishable here. This results in a small downward bias of the measured translation error rate. Detected sequences were then matched to the sequences contained in the custom database file. Some detected sequences did not have an exact sequence match because they did not conform to the predicted trypsin digestion fragmentation. This was caused by either the *in vivo* mistranslation events of the trypsin recognition amino acids (K, R, or P), or via collision-induced dissociation within the mass spectrometer. In all likelihood, those peptides named non-standard products (NSPs) were derived from mistranslation events of the trypsin-related amino acids. Proteome Discoverer performs *in silico* enzymatic digestion on-the-fly during the search process because it treats each pre-trypsinized peptide as a full-length protein. Therefore, the supplied custom database file included all possible mistranslation events that would affect trypsin cleavage; Proteome Discoverer generated those products and searched for them. Not all newly generated trypsin sites will result in two detectable peptides, as the location of the trypsin site can result in an asymmetrical cleavage that results in a peptide that falls below detection limits. Detected NSPs are then mapped back to their parent peptides during the execution of the pipeline. An internal homology filter for NSPs is also implemented to exclude NSPs that also happen to map to an internally homologous protein region. Once all detected sequences have been mapped to their peptide identifiers (peptide identifiers include the substitution type), then peptides belonging to a set of specific substitutions were filtered out due to chemical artifacts (Table 1).

**Table 1.** Chemical modifications designated as artifacts

Substitution	Monoisotopic mass	Average mass	Mass shift	Modification
Asn **→** Asp	0.984	0.985	1	Deamidation
Gln **→** Glu	0.984	0.985	1	Deamidation
Glu **→** Ser	−42.011	−42.037	−42	Unknown
Ser **→** Asp	27.994	28.010	28	Formylation
Thr **→** Glu	27.994	28.010	28	Formylation
Ser **→** Ala	−15.994	−15.999	−16	Deoxidation
Tyr **→** Phe	−15.994	−15.999	−16	Deoxidation

Due to identical mass deltas, these substitutions are indistinguishable from chemical modifications that may occur either naturally or through sample preparation for mass spectrometry.

Values shown in Table 1 were obtained from Unimod, a database of protein modifications for mass spectrometry [20]. Among the artifacts listed here is Glu–Ser, which did not have a modification within 0.02 Da of the substitution mass delta listed in Unimod, though the observed data highly suggest that this mass delta is being caused chemically. For reference, when including that substitution in the data analysis, Glu experienced an unusually high error rate of 1.5% with 81% of substitutions mapping to Ser. For comparison, when Glu to Ser is treated as an artifact (removed from analysis), then the measured translation error rate of Glu falls back into the previously reported range of codon error rates at ~3.00 × 10^−3^. Lastly, the overall error rate was calculated as the total number of detected substitutions divided by the total number of sites sampled. Error rates according to substitution type were calculated similarly, with the number of detected substitutions for each source amino acid divided by the total number of sites sampled for each source amino acid.

### Generation of substitution spectra

Both amino-acids and codon-level substitution spectra were generated during the execution of the custom pipeline. Each mutant peptide has an identifier associated with it that denotes the wild-type amino acid and the substituted amino acid. Provided each identifier and the number of PSMs associated with each peptide sequence, a number of specific substitutions was determined for each amino-acid pair in a 20 × 19 matrix, wherein 19 represents the destination amino acids where Ile and Leu have been combined into a single Xle row. This matrix represents the substitution spectra associated with detected mistranslation events. Substitutions identified as artifacts, due to indistinguishable mass differences between certain amino-acid substitutions and chemical modifications, were excluded from the analysis. The error rate per amino acid was calculated by taking the number of observed mistranslation events per amino acid and dividing it by the total number of codons sampled for that amino acid. Together, these matrices report the likelihood of a mistranslation event per amino acid, and the bias toward the incorporation of any other amino acid given a mistranslation event. The codon substitution spectrum was determined in a way very similar to the amino-acid substitution spectrum. In any detected mistranslated peptide, the exact DNA coordinates were determined, and therefore the codon that was mistranslated was determined. This allowed for the generation of a matrix that would reveal any bias in specific codons toward specific amino acids.

### Identification of significantly enriched substitutions

To determine which types of translation errors occurred more frequently than expected by chance, we performed simulations to generate a randomized dataset for comparison. 10 000 rounds of simulation were performed to generate pseudo datasets based on the marginal distributions of amino-acid sources and destinations. The marginal probability of an amino acid serving as a source or destination was defined by the proportion of the total counts per the amino-acid source or destination divided by the total number of observed mistranslation events (*n*_wild type_ = 6692). In each round of simulation, pairs of source and destination amino acids were drawn with the function *sample* in R according to the marginal probabilities, but those deemed as artifacts (E-to-S, N-to-D, Q-to-E, S-to-D, S-to-A, T-to-E, and Y-to-F), or those with the same amino acid (including I-to-X and L-to-X) were discarded. The drawing and discarding step continued until a total of 6692 pairs were acquired, which generated a pseudo dataset for a simulation round. After performing 10 000 rounds of simulation, for each substitution, the nominal *P*-value was determined by the proportion of pseudo datasets where the simulated observed numbers were larger than or equal to the empirically observed number. Given that only 10 000 rounds of simulations were performed, the case where no simulated observed value was larger than or equal to the empirically observed number was noted as *P* < .001. Under Bonferroni’s multiple testing correction (353 comparisons) and 5% family-wise error rate, only the nominal *P*-values ≤.001 were considered statistically significant. Using 10 000 simulated numbers per substitution, the mean and standard deviation were calculated. The *Z*-score for a substitution was then determined by the difference between the simulated mean and the empirical value, normalized by the simulated standard deviation.

### Abundance analysis

Protein abundance data were obtained from PaxDb integrated datasets [21]. These datasets are downloaded with non-universal protein identifiers, which requires some ID linking between parsed proteomes obtained from the custom parsing program used here. The custom parser results in proteomes with unique identifiers, such as locus tags or protein IDs. As such, a custom script was made that links PaxDb identifiers to the IDs found in the parsed proteome file by comparing protein sequences found in local files, or by fetching protein sequences from NCBI. The custom script optionally accepts a proteome file that utilizes the PaxDb identifiers as FASTA headers. In this case, the PaxDb identifiers were from the STRING database [22], so the *E. coli* reference proteome was downloaded from STRING. The custom script was executed with the STRING reference proteome and the proteome generated from the custom parser. Protein sequences in these two files were compared, if they were found to be identical then the parsed ID was linked to that PaxDb identifier. Following assignment of exact matches, PaxDb IDs that still lacked a linkage to the parsed proteome were subjected to pairwise alignment of all other protein sequences in the parsed proteome. If the alignments yielded a single match with >95% identity, then the parsed ID would be linked to the PaxDb ID. Alignments that fell between 90% and 95% identity were manually reviewed. Following the local search, STRING IDs were converted to gene symbols by retrieving the preferred names from STRING; preferred names were confirmed or revised using a public gene annotation web-service, MyGene.info [23]. These gene symbols were used to programmatically retrieve protein sequences from NCBI. Protein sequence retrieval from NCBI first uses gene symbols obtained from MyGene.info, then if any IDs remained unlinked, PaxDb preferred names are then used. These sequences were used as described before, by first searching for exact matches among the annotated protein sequences, followed by alignments. If a gene symbol listed in the PaxDb database remains unlinked, it is assumed that the corresponding protein was either not present in the annotation file or did not meet the minimum identity threshold of 90%. The vast majority of proteins had sequence identity matches >98%. Once all possible links have been made between the annotated proteome and the abundance data, then a custom script is executed for abundance analysis. This script finds all peptides that belong to a single protein in the analyzed data, then counts all wild-type and mutant amino acids for that protein. Peptides that could map to multiple proteins are removed from the analysis. The error rate of the protein is determined by taking the sum total of amino-acid substitutions for that protein, then dividing that value by the total number of amino acids sampled for that protein. Only proteins that met a minimum requirement of 3000 sampled amino acids were considered for further analysis.

### Identification of orthologs

To measure codon frequencies in the *E. coli* genome and to identify conserved and variable amino-acid sites for Akashi’s test of translation accuracy, orthologs of the proteins annotated in the wild-type strain were identified in a collection of 232 other *E. coli* genomes, including the K-12 MG1655 reference strain. These genomes were selected from a random sample of 1000 genomes from NCBI after filtering out highly similar genomes, using PopPUNK [24] and lineages that appear genetically disconnected with the rest of the population, using PopCOGenT [25]. The ortholog search was performed using a reciprocal best-hit approach with BLASTp [26], excluding hits with <80% query aligned and log2-fold difference in subject and query length exceeding 0.5. Protein accessions for the orthologs of the wild-type strain identified across this collection of *E. coli* isolates are provided in Supplementary Data File S1. Amino-acid alignments were generated using MUSCLE (v3.8.1551) and codon alignments were generated using PAL2NAL (v14) [27, 28]. For the proteins present in at least 220 out of 233 strains (∼ 95%) i.e. “core” proteins, frequencies of 61 codons at each site, excluding the first 50 and the last 20 sites, were determined.

### Identification of preferred codons

To identify preferred codons, we analyzed coding sequences of a set of 429 highly abundant proteins (top 25% proteins based on protein-abundance data from PaxDB) [21]. Preferred synonymous codons for different amino acids in *E. coli* were identified based on population-genetic analysis of codon frequencies. The Li–Bulmer model of mutation-selection balance was applied to the observed frequencies of codons at fixed sites while accounting for the effect of mutation biases on codon usage bias [29, 30]. Codon sites were considered fixed if the most frequent codon was present in at least 98% of the sequences, based on the multiple sequence alignments of protein-coding genes generated above. The relative mutation rates among 4 nucleotides were derived from the mutational spectrum of the *E. coli* strain PFM2 wild type obtained through a mutation accumulation experiment [31]. We also identified a second set of preferred codons by applying the same model to codon frequencies across all genes.

The original Li–Bulmer model provides an expression for the equilibrium probability of one of the two alleles under mutation-selection balance, *P* = 1/(1+βe^−*S*^), where *S = 2 N*_e_  *s* with *N*_e_ being the effective population size and *s* being the selection coefficient for the target allele, and β is the mutation bias toward the other allele. A four-allele extension of this model was applied to the frequencies of four codons in a codon group (defined by the first two codon positions). The construction of this model can be understood by noting that the above equation represents a solution to the stationary distribution of a two-state Markov chain with transition matrix, *P* = $${\begin{array}{@{}*{2}{c}@{}} \big[{1 - {{\mu }_{12}}{{f}_{12}}}&{{{\mu }_{12}}{{f}_{12}}}\\ {{{\mu }_{21}}{{f}_{21}}}&{1 - {{\mu }_{21}}{{f}_{21}}} \big] \end{array}}$$ where *μ*_ij_ is the mutation rate from allele *i* to allele *j* and ${{f}_{ij}} = \frac{{1 - {{\mathrm{ e}}^{ - {{S}_{ij}}{{p}_0}}}}}{{1 - {{\mathrm{ e}}^{ - {{S}_{ij}}}}}}$ is the fixation probability of *j* over *i* and *S*_ij_ = 2 *N_e_ s*_ij_ is the corresponding population-scaled selective advantage, with *p*_0_ being the initial population frequency of allele *j*. The stationary distribution, π = (*p,q*) that satisfies π = π*P*, can be found by solving a linear system of two equations under the constraint that *p* + *q* = 1 [32]. The solution for the above matrix is $\frac{1}{{{{\mu }_{12}}{{f}_{12}} + {{\mu }_{21}}{{f}_{21}}}}( {{{\mu }_{21}}{{f}_{21}},{{\mu }_{12}}{{f}_{12}}} )$. The Li–Bulmer equation can be recovered by replacing *μ*_12_/*μ*_21_ with *β*, and noting that *f*_12_/*f*_21_ equals e^−*S*^ where *S* = *S*_21_.

A four-allele model of mutation-selection balance was derived by similarly considering the stationary distribution of a four-state Markov chain. The 4 nucleotides were labeled in the alphabetical order i.e. 1 (A), 2 (C), 3 (G), and 4 (T), and mutation rates (*μ*_ij_) were set to the six base-pair substitution rates derived from the mutation-accumulation study e.g. *μ*_24_ = *μ*_31_ = *µ*_GC→AT_. The selection coefficient for nucleotide A was set to 0 and the other 3 selection coefficients (*S*_j_) were relative to A, so that the selection term (*S*_ij_) in fixation probability of allele *j* over *i* was set to


\begin{eqnarray*}
{{S}_{ij}}:= \left\{ {\begin{array}{@{}*{3}{c}@{}} {{{S}_j}}&:&{i = 1}\\ { - {{S}_i}}&:&{j = 1}\\ 0&:&{i = j}\\ {{{S}_j} - {{S}_i}}&:&{j\not= i\not= 1} \end{array}} \right..
\end{eqnarray*}

Expressions for equilibrium probabilities as functions of three selection coefficients were derived by solving the resultant system of linear equations. Values of selection coefficients were derived from these equations based on the Newton–Raphson method [32]. The Newton–Raphson approach finds roots of the vector equation *f*(x) = 0 based on iterations of the form x_t+1_ = x_t_ – (*Df*(x_t_))^−1^  *f*(x_t_), where *D*f(x) is the Jacobian matrix. In our problem, *f*(x) represents the difference between equilibrium and observed frequencies of the four codons in a group. Therefore, estimated selection coefficients correspond to values that equate the expected equilibrium frequencies under mutation-selection balance to their observed values. Starting estimates for selection coefficients were arbitrarily set to (0.1, 0.2, and 0.3). This analysis was performed in Mathematica 13.2. For amino acids with less than four synonymous codons, selection coefficients initially estimated relative to codon NNA were recalculated relative to the smallest selection coefficient among synonymous codons. Preferred codons for each of the 18 amino acids with more than one synonymous codon were identified as the one with the highest selection coefficient. For three amino acids with six synonymous codons, i.e. codons belonging to two-distinct codon groups, the preferred codon was identified from the group with the higher selection coefficient. The resulting set of preferred codons predicted (Supplementary Table S1—Column 1) matched those suggested by Sharp and Li [33] (Supplementary Table S1—Column 3) that were defined based on the observed codon frequencies of only 27 highly expressed genes, except for Ala, Ser, and Gly.

### Measure of codon usage bias

The codon usage bias of a gene was quantified as the proportion of preferred codons out of the total number of codons of an amino acid in that gene, averaged over 18 amino acids with more than one synonymous codon. This metric was used to test for the enrichment of preferred codons in highly expressed genes and was also applied to the preferred codons defined from all genes (Supplementary Fig. S1). Codon-level relative mistranslation rates (RMRs) for the four codons that differ between the two sets are reported in Supplementary Fig. S2. Codon usage bias was calculated for 1100 genes with at least 4 occurrences of 16 out of 18 amino acids with preferred codons, and at least 30 codons left after removing the first 50 and the last 20 codons.

### Akashi’s test of translation accuracy hypothesis

The enrichment of preferred codons at sites with conserved amino-acid residues was tested following the method used by Akashi 1994 [8]. Conserved amino-acid sites were defined as the sites where the most frequent amino acid was at least present among 98% of the 232 *E. coli* isolates. Non-core proteins, proteins missing from the wild-type strain, and those without an ortholog or more than one ortholog in *Salmonella enterica* were excluded. Contiguous stretches of mutations were removed, along with the first 50 and the last 20 codons of each gene. Sites with gaps, stop codons, or undetermined codons in any sequence were removed. Sites where the wild-type residues are among the 18 amino acids with preferred codons identified as above, and are the same as the most frequent amino acid at the site, were retained. 2 × 2 contingency tables of the numbers of preferred codons at conserved and variable sites in the wild-type strain were created for each of the 18 amino acids with synonymous codons in each protein. The tables with zero margin totals were excluded. The remaining tables were pooled following Mantel–Haenszel’s procedure and a *χ*^2^ test of the enrichment of preferred codons at conserved sites was performed [34]. The Mantel–Haenszel’s statistic, *W*_MH_, which is equivalent to an odds ratio (OR) for the pooled dataset, was calculated as $\frac{{\mathop \sum \nolimits_i {{a}_i}{{d}_i}/{{n}_i}}}{{\mathop \sum \nolimits_i {{b}_i}{{c}_i}/{{n}_i}}}$, where *i* indexes a 2 × 2 contingency table corresponding to an amino acid in a gene, *a_i_* and *b_i_* stands for the number of preferred and unpreferred codons, respectively, at conserved sites, and *c_i_* and *d_i_* stands for the number of preferred and unpreferred codons, respectively, at variable sites, and *n_i_*= *a_i_*+ *b_i_*+ *c_i_*+ *d_i_* is the total number of sites considered for that amino acid in that gene. The *χ*^2^ test-statistic was calculated, with a continuity correction, as


\begin{eqnarray*}
\frac{{{{{( {| {\mathop \sum \nolimits_i {{a}_i} - \mathop \sum \nolimits_i ( {{{a}_i} + {{b}_i}} )( {{{a}_i} + {{c}_i}} )/{{n}_i}} | - 0.5} )}}^2}}}{{\mathop \sum \nolimits_i ( {{{a}_i} + {{b}_i}} )( {{{a}_i} + {{c}_i}} )( {{{b}_i} + {{d}_i}} )( {{{c}_i} + {{d}_i}} )/( {n_i^3 - n_i^2} )}}.
\end{eqnarray*}

The significance of the association between preferred codons and conserved sites was evaluated under a χ2 distribution with one degree of freedom.

### Statistical analyses

Each ribosomal variant was evaluated using three biological replicates (*n* per variant = 3). Mean error rates (codon-specific or amino-acid-specific) were calculated by averaging across the three replicates per variant. Differences in overall translation-error rates, position-specific misreading, and minimum mutational distance (MMD) were evaluated by one-way ANOVA (two-sided). RMRs of preferred codons were tested against a null of 1 using a two-sided one-sample Wilcoxon signed-rank test across the 18 amino acids with synonymous codons. The standard error for each codon RMR was calculated by propagation of errors, which takes into account the standard error of each codon’s mean error rate (the RMR numerator) and the standard errors for the codons comprising the mean error rate for the synonymous codon family (the RMR denominator). Given that $\bar{e}$ is a mean error-rate, the propagation of errors formula for RMR is


\begin{eqnarray*}
\mathrm{ S}{{\mathrm{ E}}_{\mathrm{ RMR}}} = \mathrm{ RMR} \times \sqrt {{{{\left[ {\frac{{\mathrm{ SE}\left( {{{{\bar{\mathrm{ e}}}}_{\textrm{codon}}}} \right)}}{{{{{\bar{\mathrm{ e}}}}_{\textrm{codon}}}}}} \right]}}^2} + {{{\left[ {\frac{{\mathrm{ SE}\left( {{{{\bar{\mathrm{ e}}}}_{\textrm{family}}}} \right)}}{{{{{\bar{\mathrm{ e}}}}_{\textrm{family}}}}}} \right]}}^2}} .
\end{eqnarray*}

A paired one-sided Wilcoxon signed-rank test was used to compare RMR values between high-abundance and low-abundance proteins because our analysis up to that point suggested that preferred codons in highly expressed genes were less accurate. In addition, RMRs in each expression group were tested against a null of 1 using two-sided one-sample Wilcoxon signed-rank tests. Spearman’s rank correlation test was used to test for an increase in frequency of preferred codons with protein abundance, independent of assumptions of a linear regression. All statistical tests were performed in R (v4.4.1), Python (v3.12), and Mathematica (v13.2).

## Results

### Amino-acid substitution spectra reveal bias in mistranslation events

**Figure 1.**
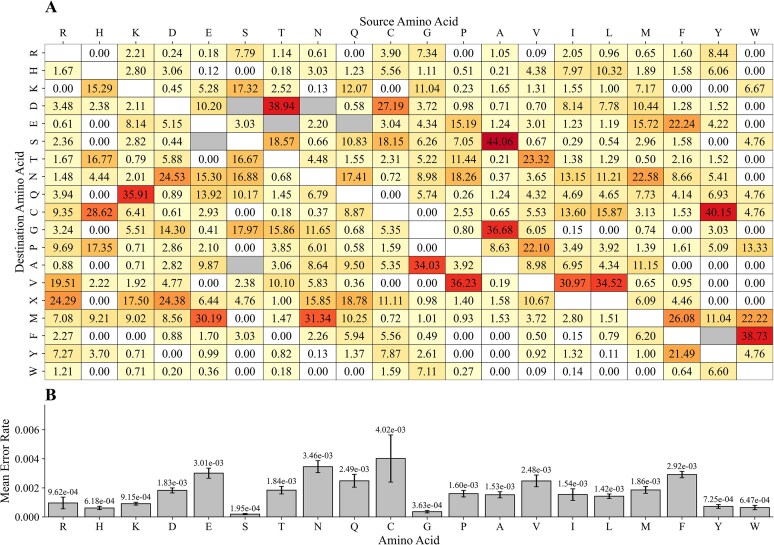
Mean rate and spectrum of translation errors of wild-type *E. coli*. (**A**) Wild-type (source) amino acids are represented as columns, and erroneously incorporated (destination) amino acids are represented as rows. (**B**) The mean rate at which each amino acid is mistranslated is plotted with standard error bars across three biological replicates. Substitutions that are indistinguishable from chemical modifications of the source amino acid (see Table 1) are removed from the analysis (gray boxes). Leucine and isoleucine are combined in the destination row “X.” Panel (B) gives the average rate at which each amino acid is mistranslated, while panel (A) gives the percentage distribution of those mistranslations across destination amino acids.

We estimated translation-error rates and spectra of each ribosomal variant believed to differ in their translational fidelity (Fig. 1 and Supplementary Figs S3 and S4). The mean translation-error rate for the wild type (1.82 × 10^−3^ per codon, SE = 5.92 × 10^−5^) was similar to the putative accurate ribosomal variant (1.86 × 10^−3^, SE = 1.83 × 10^−4^). In comparison, the putative error-prone ribosomal variant exhibited a mean error rate ~20% higher (2.23 × 10^−3^, SE = 2.19 × 10^−4^). However, the differences in overall translation-error rates among the three variants were not statistically significant (one-way ANOVA, *P* = .17). While the proteome-wide error rate estimates do not recapitulate the previously described fidelity differences observed in single-codon assays, the error spectra reveal variant-specific mistranslation profiles according to substitution type and codon.

To characterize the spectrum of translation errors in wild-type *E. coli*, we identified substitutions occurring significantly more often than expected given a simulated distribution of expected substitutions based on the marginal probabilities of each substitution type. Source and destination amino acids were paired independently during 10 000 rounds of simulation according to their observed frequencies among all detected errors (the “Materials and methods” section). A *χ*^2^ analysis comparing the observed and simulated data revealed that mistranslation rates vary significantly according to substitution types (*χ*^2^ = 1585.63, *P* < .001) (Supplementary Tables S2–S4). Notably, serine and glycine exhibited significantly lower error rates compared to other amino acids, while glutamic acid and asparagine experienced significantly higher error rates. The molecular details in the collected data also allow us to quantify the error rates per codon (Supplementary Figs S5–S7), or per protein.

### Position-specific decoding fidelity differs among ribosomal variants

**Figure 2.**
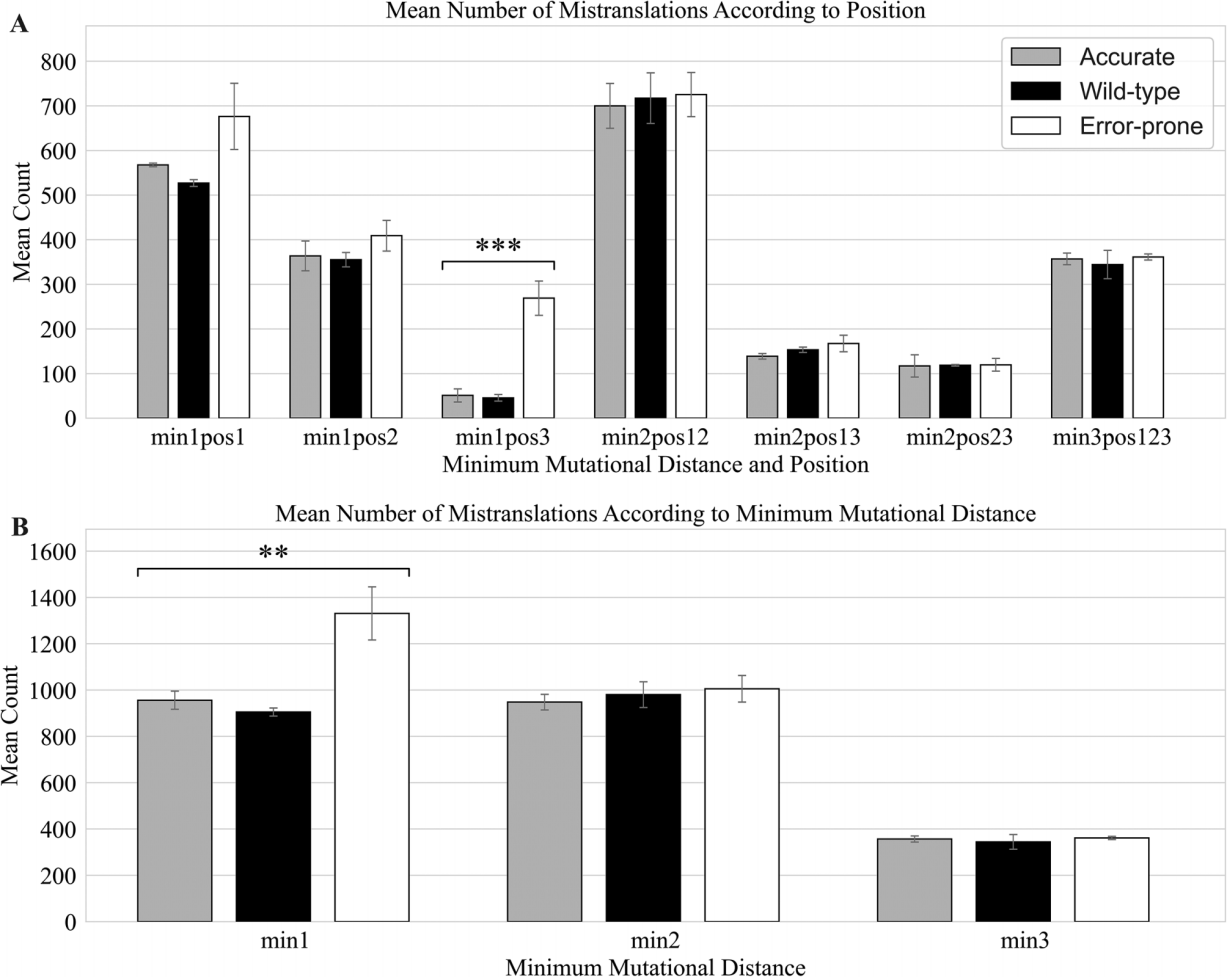
Mistranslation events according to MMD and position. (**A**) Each ribosomal variant is color-coded according to the legend. The categories start with “min” followed by a number, indicating the MMD. For each MMD, the position is indicated by “pos” followed by the number(s). Codon position is left to right, 1–3. Error bars represent the standard error of the mean. Asterisks are indicative of statistically significant differences, **P ≤ .01 and ***P ≤ .001. (**B**) Mean number of mistranslation events according to MMD.

Decoding errors can be caused by ribosomal misreading at any of the 3 nucleotide positions within a codon. Inferring the likely position of each error, based on the nature of the genetic code, provides insight into position-specific decoding fidelity. The top 5% of enriched substitutions according to *Z*-score (Supplementary Tables S5–S7) were classified according to the minimum number of within codon positions needed to be misread to produce the observed substitution (MMD). The MMD provides insight into the mechanism of mistranslation, such as likely ribosomal misreading events (MMD = 1) from probable tRNA mischarging (MMD = 3) (complete data in Supplementary Tables S2–S4). The putative error-prone ribosomal variant misreads codons at the third position significantly more often than the wild-type or accurate ribosomal variants (*P* = .0009, one-way ANOVA; Fig. 2A). Additionally, when missense substitutions occur, the MMD of the error-prone ribosomal variant is significantly more likely than the wild-type or accurate ribosomal variants to be only 1 nucleotide (*P* = .01, one-way ANOVA; Fig. 2B).

AAU asparagine has been widely used as a near-cognate challenge in luciferase reporter assays because it differs by only a single nucleotide from AAA lysine (the wild-type codon at the active site of luciferase). In fact, the *E. coli* ribosomal variants used in this study were previously characterized via luciferase reporter assay [3, 9]. We find that the error-prone variant has an AAU error rate approximately double that of the other two ribosomal variants (5.62 × 10^−3^ versus 2.99 × 10^−3^ and 3.30 × 10^−3^). More importantly, 52% of AAU mistranslations in the error-prone variant were to lysine (Supplementary Fig. S7), whereas lysine was never erroneously incorporated at AAU codons for the wild-type and accurate variants (Supplementary Figs S5 and  S6). Thus, the combination of a higher error rate at the AAU codon and a bias toward incorporating lysine—the amino acid enabling luminescence in the reporter assay—likely led to prior overestimates of the translation-error rate of the error-prone variant. Even when third-position AAU mistranslation events are removed from the analysis, third-position errors remain significantly elevated in the error-prone variant relative to the other ribosomal variants (*P* = .003, one-way ANOVA), indicating that the difference cannot be attributed to AAU mistranslation alone.

### Codons favored in highly expressed genes are translationally inaccurate

**Figure 3.**
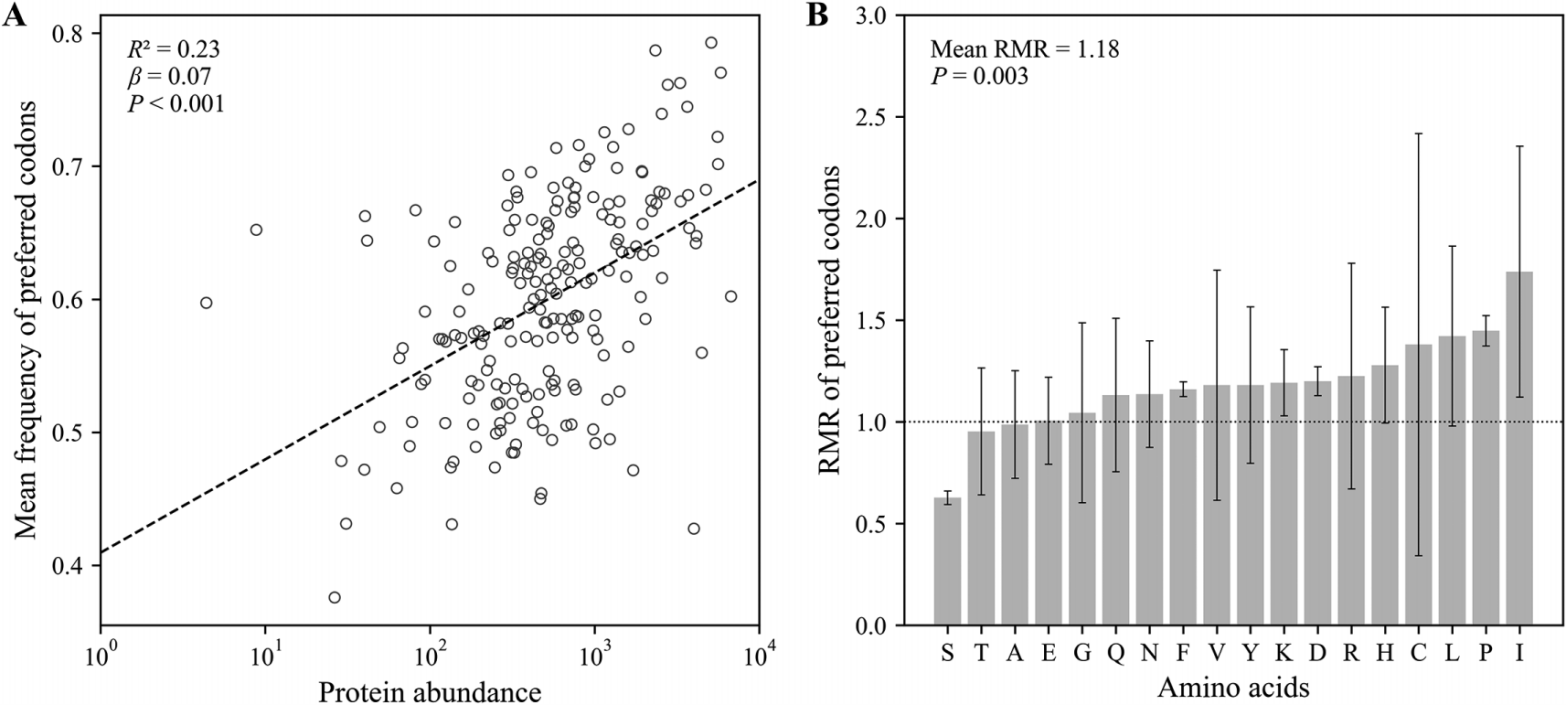
Translational accuracy of codons favored in highly expressed genes. (**A**) Enrichment of preferred codons in highly expressed genes. The enrichment is measured as the relative frequency of preferred codons among synonymous codons in a gene averaged over amino acids. The linear regression coefficient between this measure of codon usage bias and log protein abundance for 205 genes (data from PaxDb) is *β* = 0.070 (SE = 0.009) and the Spearman rank correlation coefficient is *ρ* = 0.50 (*P* = 3.51 × 10^−14^). (**B**) RMR of preferred codons, for the 18 amino acids with more than one synonymous codon. RMR > 1 indicates higher error rates for preferred codons compared to the mean error rate of their respective synonymous codon groups. The height of each bar corresponds to the mean RMR of each preferred codon over three biological replicates. The mean RMR across the 18 preferred codons is 1.18 and significantly >1 (one-sample Wilcoxon signed-rank test, *P* = 3 × 10^−3^). Error bars denote the corresponding standard errors of the mean.

Synonymous codons are used unequally across the *E. coli* genome. Frequencies of some codons increase with gene expression, and correlate with the cellular abundance of their cognate tRNAs, leading to the suggestion that these codons are favorable to the process of translation [35–37]. The standard model of “translational selection” considers that codons are preferred for their translational speed as well as accuracy [38]. However, a direct test of this hypothesis has been lacking due to the absence of genome-wide, codon-level translation-error rate estimates. Because the intrinsic mutational biases of a genome also shape its codon composition, the most frequently used codon for an amino acid is not necessarily favored by selection [37, 39]. We therefore identified preferred codons based on a quantitative model that takes mutational biases into account (the “Identification of preferred codons” section) [29, 30] (Supplementary Table S1). We found a strong positive correlation between the average frequency of preferred codons among synonymous codons in a gene and the abundance [21] of its protein product (Fig. 3A). Furthermore, the relative expression of cognate tRNAs [40, 41] was greater for preferred codons compared to that of their synonyms (Supplementary Fig. S8), which is consistent with translational selection.

With the codon-level mistranslation rates estimated in this study (Supplementary Fig. S9), we can directly test whether codons preferred under translational selection make fewer translation errors. We compared mistranslation rates of preferred codons with that of their non-preferred synonymous codons. For each codon of an amino acid, we calculated an RMR, defined as the ratio of the mistranslation rate for a codon and the mean mistranslation rate of all synonymous codons for that amino acid [42]. For example, the RMR of the Pro codon CCA is calculated by taking the mean error rate of the CCA codon, 1.71 × 10^−3^, and dividing it by the average of all Pro codon error rates (CCA, CCC, CCG, CCU), 1.60 × 10^−3^, resulting in an RMR of 1.06 for the CCA codon. An RMR >1 indicates that a codon is less accurate than an average codon for the same amino acid. The mean RMRs of preferred codons over three biological replicates were significantly different from 1 (two-sided Wilcoxon signed-rank test, *P* = .0028) (Fig. 3B). Serine was the only amino acid with a mean RMR for the preferred codon significantly <1. However, for no amino acid was the preferred codon the most accurate one. Thus, we find that codons thought to be favored by translational selection are actually more susceptible to mistranslation.

The translation-accuracy hypothesis for codon usage states that codons favored in highly expressed genes should be translated more accurately compared to other synonymous codons. Previous studies have found support for this hypothesis in several organisms, including *E. coli*, based on an indirect approach proposed by Akashi [8, 13, 42, 43]. Akashi predicted that mistranslation errors would be most consequential for functionally critical amino-acid residues; therefore, codons selected for translational accuracy should be enriched at evolutionarily conserved sites. Because we see that codons favored in highly expressed genes are translated less accurately, we expect these codons to be less frequent at conserved sites. To test this expectation, we applied Akashi’s test, which compares the frequency of preferred codons at conserved versus non-conserved sites across orthologs, to our predicted set of preferred codons using a collection of 232 *E. coli* genomes (sampled from NCBI genome database; see the “Materials and methods” section). We excluded certain groups of sites known to introduce artifacts [43], and calculated OR of counts for preferred and non-preferred synonymous codons at conserved and variable sites, such that an OR > 1 indicates an enrichment of preferred codons at conserved sites (the “Materials and methods” section). Preferred codons were neither enriched nor underrepresented at conserved sites (OR = 0.98, *χ*^2^ = 0.60, *P* = .438), suggesting that translation accuracy has played a limited role in shaping codon preference of highly expressed genes in *E. coli*.

### Protein-level error rates are positively correlated with protein abundance

**Figure 4.**
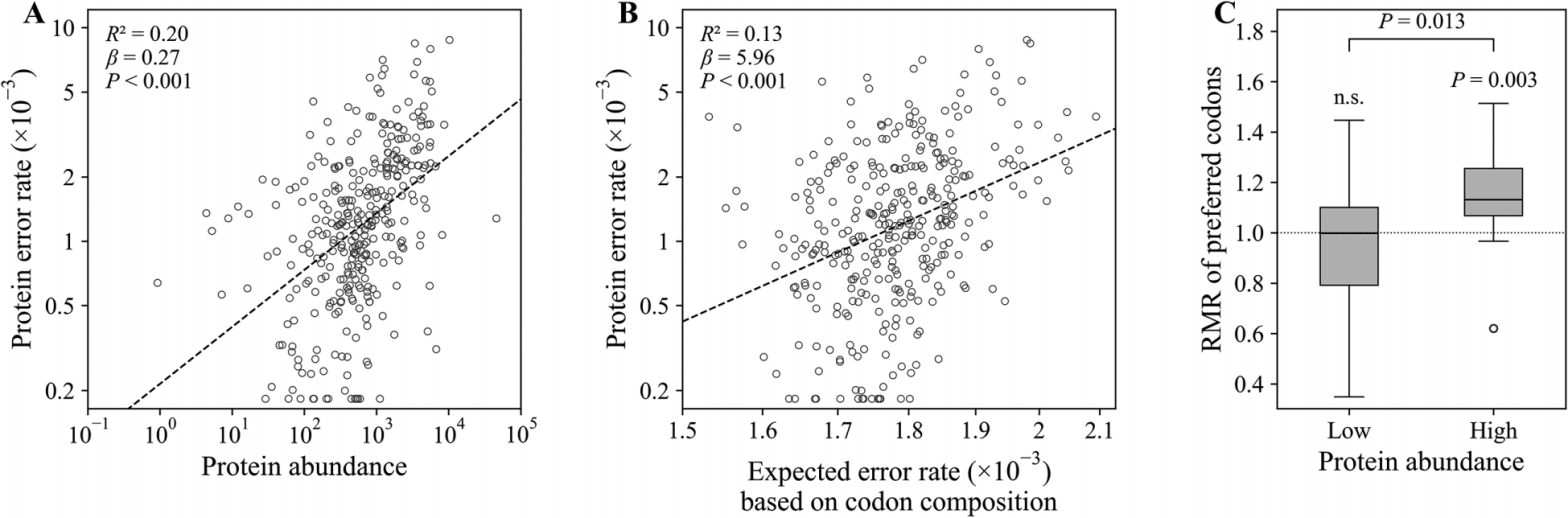
Protein abundance and codon composition as predictors of protein mistranslation rates. (**A**) Observed error-rates of proteins are positively correlated with protein abundance. Linear regression coefficient for log-transformed values, *β* = 0.27, *R*^2^ = 0.20, *P* = 9.1 × 10^−18^. This analysis was performed with 332 proteins with protein abundance data (PaxDB) and at least 3000 observed codons in the mass-spectrometry data. (**B**) Observed protein error rates are correlated with the expected error rates based on coding sequence. Linear regression coefficient, *β* = 5.96, *R*^2^ = 0.13, *P* = 1.5 × 10^−11^. (**C**) Estimates of RMRs of preferred codons differ between expression levels. Low and High represents the bottom 50% and top 50% of 1714 genes, respectively, based on protein abundance data. Boxplots are drawn over RMRs of preferred codons for 18 amino acids. RMR values for Low and High were tested against a null hypothesis of 1 (two-sided one-sample Wilcoxon signed-rank test, *P =* .35 and *P* = .003, respectively). Boxes indicate the interquartile range with the median shown as the center line. Outliers that are farther than 1.5 times the interquartile range outside the box are shown separately. The dashed horizontal line at RMR = 1 indicates the expectation that preferred codons do not differ from other synonymous codons in translation accuracy.

A key prediction of the translational-accuracy hypothesis is that highly expressed genes will evolve to be translated more accurately due to the negative fitness effects associated with mistranslating a highly abundant protein [13, 44]. Contrary to this expectation, we observed high error rate estimates for codons enriched in highly expressed genes (Fig. 3), which would suggest that highly-expressed genes are more error-prone. To determine whether the translation accuracy of a protein is governed by its sequence or expression level, we tested the correlation of protein error rates with protein abundance and codon composition. The error-rate of a protein is the proportion of mistranslated amino acids among all amino acids mapped to the protein in the mass-spectrometry data. Contrary to the prediction of the translation-accuracy hypothesis, we found that protein error rates were indeed positively correlated with protein abundance (Fig. 4A). Next, to test the effect of codon composition on translation accuracy of a protein, we defined an expected error rate for a protein based on the average error rate of all codons in the underlying gene sequence. Observed protein error rates correlated with the error rates expected based on codon composition (*R*^2^ = 0.13, *P* = 1.45 × 10^−11^) (Fig. 4B). A multiple linear regression including both protein abundance and expected sequence-based error rates slightly improved predictive power (*R*^2^ = 0.23, *P* < .001). Both predictors were significant (*P* < .001), though abundance was a stronger predictor (standardized coefficients: 0.36 versus 0.20). Consistent with this, preferred codons were less accurate in highly abundant proteins than in lowly abundant proteins (Fig. 4C, *P* = .013, one-sided Wilcoxon signed-rank test), indicating that translational inaccuracy is more pronounced at higher expression levels.

## Discussion

We have presented a method that estimates both the rate and spectra of translation errors. Making use of historically characterized ribosomal variants of *E. coli* to verify the method, we had expected translation-error rates to reflect their originally assigned phenotypes: wild type, accurate, and error-prone [3, 10, 11]. However, when considering nearly the entire spectrum of possible mistranslation events, as opposed to a single codon in a single protein, the overall error rates of these variants did not significantly differ from one another. What did change were the types of mistranslation events that were enriched in each variant (Supplementary Tables S5–S7). The differences among the mistranslation profiles were likely related to either kinetic or structural differences specific to each ribosome. The “error-prone” ribosomal variant struggled to accurately discriminate near-cognate codons that differed in the 3rd position, which is consistent with previously reported kinetics suggesting that the error-prone variant fails to reject near-cognate tRNA at a rate ~10-fold higher than wild type [3]. The impact of this position-specific misreading is limited due to the degeneracy of the genetic code. The opportunity for error in the third position represents a small fraction of all single nucleotide errors; of 415 possible errors due to single nucleotide misreads, only 57 involve the third position. As such, the putative error-prone variant did exhibit an overall error rate 20% higher than wild type, but this difference was not statistically significant.

Mass-spectrometry acquisition strategies generally fall into two categories: data-independent acquisition (DIA), which systematically fragments all peptides (precursors) within an isolation window, or DDA, which selects individual precursors based on abundance for fragmentation and sequential fragment ion scanning. Because DIA fragments multiple peptides at the same time, the resulting chimeric spectra are challenging to deconvolute even for abundant peptides; detecting low abundance mistranslated peptides would face significant signal-to-noise limitations. In contrast, DDA generates cleaner fragment spectra for individual precursors, making it better suited for identifying rare mistranslated peptides. In DDA mode, an initial survey scan of intact peptides (MS1) is followed by fragmentation of the most abundant precursors, generating fragment ion spectra (MS2) from which peptide identities are determined. In order to avoid redundant sampling of the most abundant peptides, a dynamic exclusion window temporarily prevents resampling of a precursor after its MS2 spectrum has been acquired. Translation-error rates can be estimated either from MS1 signal intensities [45] or from spectral counts [46, 47], the number of times a peptide was independently selected for fragmentation. Because instrument settings such as dynamic exclusion duration, MS2 acquisition mode, and resolution all affect which peptides are detected, as well as how often, these parameters must be controlled when comparing error rates across experiments.

Comparing our results to a recent spectral-counting approach [46, 47] reveals substantial differences. These studies have reported an *E. coli* translation error rate of 8.99 × 10^−5^, with codon-specific rates primarily ranging from 10^−5^ to 10^−4^ [46, 47]. These reported rates are far lower than any previous estimate of translation error rates, which have historically been reported in the range of 10^−4^–10^−3^ (see Kramer and Farabaugh [9] and references therein). They are also low enough to overlap with reported transcription error rates (10^−6^–10^−5^) [48, 49]. If translation error rates are indeed as low as reported, then it raises the question of whether the measured signal is reflective of translation errors or transcription errors. In contrast, we report a wild-type *E. coli* translation error rate of 1.82 × 10^−3^, with codon-specific rates generally ranging from 10^−4^ to 10^−3^, consistent with the previously established range. The difference in error rate estimates is likely due to the aggregation of 80 public datasets in the prior study [46], which introduced uncontrolled variability in acquisition parameters, sample biology, and genetic background.

Just 3 of the 80 datasets accounted for ~60% of all detected errors. Among the top five contributing studies [45, 50–53], representing approximately one-third of the total data, dynamic exclusion durations ranged from 10 to 60 s. Short dynamic exclusion windows lead to oversampling of the same abundant wild-type peptides. This repeated sampling of wild-type peptides inflates wild-type PSM counts relative to error-containing PSMs, depressing the estimated error rate. Although only one of these five studies used a B-strain *E. coli* (while the others made use of K12 derivatives), the use of a single K12 reference proteome would bias error rates toward an overestimate due to strain-specific polymorphisms between K12 and B-strain *E. coli*. These polymorphisms would lead to misclassification of wild-type peptides as errors when they fail to match the reference sequence. Given that only one study among the top five data-contributing studies in the aggregate dataset made use of a B-strain *E. coli*, the dominant bias appears to be the oversampling of K12 wild-type peptides due to short dynamic exclusion durations, which systematically depresses the error rate.

These prior studies also included conditions that can alter translation fidelity. Some examples include auxotrophic strains under amino-acid starvation conditions. Those strains would experience elevated error rates due to charged tRNA competition. Strains undergoing protein overexpression can have quality control mechanisms overwhelmed and deplete certain tRNAs. One study alone included data from over 20 growth conditions across three strains. These analyses were not restricted to wild-type strains in standard conditions, but included many strains across numerous conditions compared to a single proteome. Pooling data from various strains and conditions conflates condition-dependent variation with the baseline error rate. Our estimates were obtained from *E. coli* grown at 37°C and collected at stationary phase. Protein expression profiles and post-translational modifications can vary with growth conditions [52], and translation accuracy may behave similarly, though this has not been directly tested here.

Previous studies examining translation-error rates via mass spectrometry have controlled for PSMs at a 1%–5% FDR [45–47]. Though this may be standard for protein identification, it may be insufficient for accurate estimations of translation-error rates. FDR is a dataset-level metric, controlling the proportion of false positives across all identifications rather than the false-positive probability of individual matches. Abundant wild-type peptides produce spectra with higher signal-to-noise ratios. This leads to high-confidence identifications of wild-type peptides that can then lower the overall FDR of the entire dataset. This is problematic because it allows false-positive, low-abundance peptides to pass the FDR threshold into the filtered dataset. This is particularly concerning because confident identification of low abundance, error-containing peptides is critical for determining accurate translation error rates. In this study, we make use of the PEP score, as it ensures a true 1% error probability per individual spectral match [54].

These considerations for controlling variability have motivated both the direct database search strategy and the controlled acquisition parameters for obtaining our LC-MS/MS data. In this study, these variables were controlled by acquiring all data on a single instrument with consistent high-resolution acquisition parameters, and by using defined strains grown under standard lab conditions. It should be noted that spectral counting has been used as a semi-quantitative measure of protein abundance in aggregate [55–58], but PSM counts for individual peptides may not reflect their true individual abundance. Single amino-acid differences between otherwise identical peptide pairs can alter chromatographic behavior and ionization efficiency due to changes in physicochemical properties [59, 60], meaning PSM counts for a given peptide pair may not be directly comparable with each other. We mitigate this limitation by aggregating spectral counts across all observed instances of a given error type (e.g. amino-acid-to-amino-acid and codon-to-amino-acid). Overall translation error rates are similarly calculated by taking the sum of detected errors over the total number of sites sampled. This approach assumes that peptide-level biases in detection of individual peptide pairs will average out over many independent observations of the same error type, but this assumption has not been experimentally validated here or previously.

For the wild-type ribosomal variant in this study, the most significantly enriched substitution was alanine to serine. Ala to Ser substitutions have been previously shown to be caused by tRNA mischarging [6, 61]. The alanine tRNA synthetase is unique in that it can mischarge with amino acids that are both larger and smaller than its cognate amino acid, and as such there is also enrichment for the other common Ala mischarging event, Ala to Gly. In fact, alanine tRNA mischarging is so pervasive across the Tree of Life that additional genomic elements have evolved to proofread charged alanine tRNA [7, 62]. The previously estimated rate of mischarging by the alanine synthetase in *E. coli* of 2.00 × 10^−3^ [6, 7, 61] is approximately equal to the alanine-specific error rates observed here for each of the ribosomal variants, which are 1.53 × 10^−3^ for wild type, 1.80 × 10^−3^ for the accurate variant, and 2.11 × 10^−3^ for the error-prone variant. Additionally, only a single nucleotide needs to be misread for alanine to be substituted by either serine or glycine. This combination of high mischarging rates and short mutational distance leads alanine mistranslation events to predominantly result in the misincorporation of serine and glycine. Short mutational distances and tRNA mischarging activity explain many of the observed enriched substitutions [6, 7, 61, 63–67]. For enriched substitutions Thr to Asp (MMD = 2) and Phe to Glu (MMD = 3), there is no prior literature of tRNA misactivation, though the MMD may suggest it.

Considering the variation in error spectra across ribosomal variants, the “accurate” variant differed from the wild type in only two substitutions—Gln to Leu/Ile and Asp to Asn, whereas the “error-prone” variant exhibited seven differentially enriched substitution types. Six of the seven differentially enriched substitution types involved MMD of 1 nt, and four of those six occurred in the third position of the codon. The ribosomal decoding center houses two conserved adenine decoding nucleotides (A1492 and A1493) that monitor the first- and second-position of the codon–anticodon complex [68]. The third position is not strictly monitored by a dedicated decoding nucleotide [68], so a ribosomal variant with reduced fidelity may be expected to accumulate errors where codon–anticodon monitoring is weakest. Here, we count only single-nucleotide misreads that change the encoded amino acid, excluding those that introduce a stop codon, which would truncate the protein rather than produce a substitution we could detect. Due to the degeneracy of the genetic code, only a small fraction of 1-nt misreads at the third position produce amino-acid substitutions (50 of 392), so the enrichment of third-position errors is unlikely to reflect substitution opportunity alone. The error-prone variant carries a truncation of ribosomal protein S4 that destabilizes its interface with S5. This structural change alters the initial selection step of translation, resulting in slower dissociation of the codon-recognition complex and accelerated GTPase activation of near-cognate tRNA complexes [3]. These changes to initial selection can produce ribosomal decoding errors at any position. Our observation that six of the seven differentially enriched substitution types in the error-prone variant corresponding to near-cognates is consistent with its prior characterization [3]. In contrast, 2-nucleotide misreads are not enriched because the tRNAs involved are non-cognate and are rejected before a stable codon-recognition complex can form.

The accurate and error-prone *E. coli* ribosomal variants were both previously classified according to the results of a reporter assay with a single nucleotide change at the third position [3]. AAU Asn is used as the experimental codon in luciferase assays because it is the near-cognate codon of Lys AAA, the wild-type amino acid in the active site of luciferase [3, 9]. However, in the present study, it was found that the error rate at AAU codons was approximately twice that of the other ribosomal variants. Additionally, mistranslation at AAU codons in the “error-prone” variant resulted in lysine misincorporation 52% of the time, whereas the other ribosomal variants never incorporated lysine at AAU codons (0%). As such, the “error-prone” ribosomal variant may have been misclassified as a consequence of a focus on one unusual codon. The patterns of mistranslation we observe are specific to each ribosomal variant. The substitutions enriched in a given variant can be attributed to known mischarging events or to short mutational distances between codons and misincorporated amino acids (e.g. Ala codons to Ser or Gly). Specific codon biases also appear uniquely according to the ribosomal variant (e.g. AAU to Lys). A systematic artifact of acquisition or database searching would instead be expected to appear uniformly across variants. Some shared error patterns do appear where the underlying biology is common, but specific errors that distinguish one variant from another cannot be explained this way. The occurrence of codon- and variant-specific biases therefore point toward the detection of genuine translation errors rather than artifacts of the method employed here.

Codon bias has long been thought to relate to either translation accuracy or translation efficiency [69–72]. Lacking direct measurements of translation accuracy, others have attempted to link synonymous codon usage to translational accuracy through applications of Akashi’s test of enrichment of preferred codons at conserved amino-acid sites [13, 43]. With direct measures of codon-level mistranslation rates obtained here, we show that codons selectively favored in highly abundant proteins are not translated more accurately and, accordingly, are not enriched at conserved sites. Stoletzki and Eyre-Walker (2006) observed a significant enrichment at conserved sites of preferred codons identified by Sharp and Li [33], but we did not observe such an association for this set of preferred codons (Supplementary Fig. S10). These differences could be due to the use of three ecologically distinct strains of *E. coli* in the Stoletzki and Eyre-Walker analysis, where it is difficult to distinguish sites that are less conserved due to weaker functional constraints from sites under divergent selection. In contrast, we analyzed hundreds of *E. coli* isolates, with an intermediate-level of divergence and evidence of ongoing gene flow, from which sites accumulating neutral variation should be more easily detectable.

We find that protein-specific translation-error rates are positively correlated with protein abundance (*R*^2^ = 0.20, *P* < .001). This finding is somewhat unexpected given prior hypotheses that abundant proteins should be translated more accurately [13, 44]. However, these prior studies have lacked direct evidence of translational accuracy; instead, accuracy was indirectly assessed using simulations to determine whether translation errors led to peptide misfolding or aggregation. Translational speed and accuracy tradeoffs have been studied in great detail, but the general thought has been that ribosomes are optimized for speed rather than accuracy [73–75]. We have shown here that certain codons are enriched in highly expressed genes beyond the frequencies expected based on mutational biases alone. Based on the data obtained in this study, we posit that for highly expressed proteins, selection favors speed of translation over accuracy. Almost all of the preferred codons (14 of 16) use either the most abundant tRNA or end in a G or C. With the exception of Val and Arg, GC-ending codons are known to be decoded faster than AT-ending codons [76]. Numerous studies have also found that abundant tRNAs enhance translational speed, and conversely that rare codons slow translation as the ribosome waits for the correct tRNA [72, 76–84].

Given the data obtained in this study, we posit that for highly expressed proteins, selection favors speed of translation over accuracy, as the fitness cost of an occasional defective protein is offset by numerous functional copies. In contrast, for proteins that are produced in low amounts, accurate translation may be more important as any defects would affect a proportionally larger fraction of the total. Indeed, the notion that translation prioritizes speed rather than accuracy has support via biophysical models, given that the level of error experienced is tolerable [38, 75]. Additionally, one kinetic model [75] described that error-rate minimization occurs at low speeds, consistent with Hopfield and Ninio [85, 86]. Our data support this, that less abundant proteins, containing more slowly decoded codons, are translated more accurately. Synonymous codons do differ in their translation accuracy but the enrichment of certain codons in highly expressed genes, albeit driven by natural selection, has not resulted in more accurate translation in *E. coli*. Although it is too early to reject the translation accuracy hypothesis based on the results of a single species, for the conditions examined here, we do not find support for it in *E. coli*.

## Supplementary Material

gkag674_Supplemental_File

## Data Availability

The method presented here for directly searching mass spectrometry data for error-containing peptides is amenable for use with existing mass spectrometry data. The code to perform this analysis is available on GitHub (github.com/LynchLab/ErrorRateAnalysis). Source code is also available at Zenodo (10.5281/zenodo.15651074). The mass spectrometry proteomics data have been deposited to the ProteomeXchange Consortium via the PRIDE [87] partner repository with the dataset identifier PXD064116 and project DOI of 10.6019/PXD064116.
