# Supplementary material for: Translation accuracy in E. coli
Source: Nucleic Acids Res. 2026 Jul 6;54(13):gkag674. doi: 10.1093/nar/gkag674 (PMC13335486; doi:10.1093/nar/gkag674)
Supplement: gkag674_Supplemental_File [file gkag674_supplemental_file.pdf]

Table S1. Selectively preferred codons as determined by the Li-Bulmer model of mutation-selection-drift equilibrium [1, 2] applied to codon frequencies in the top 25% of genes by protein abundance (PaxDb) or, alternatively, all genes.

Amino acid	(1) Top 25%	(2) All	(3) SL87
A	GCG	GCG	GCT
C	TGC	TGC	TGC
D	GAC	GAT	GAC
E	GAA	GAA	GAA
F	TTC	TTC	TTC
G	GGC	GGC	GGT
H	CAC	CAT	CAC
I	ATC	ATC	ATC
K	AAA	AAA	AAA
L	CTG	CTG	CTG
N	AAC	AAC	AAC
P	CCG	CCG	CCG
Q	CAG	CAG	CAG
R	CGT	CGC	CGT
S	AGC	AGC	TCT
T	ACC	ACC	ACC
V	GTT	GTG	GTT
Y	TAC	TAC	TAC

The four rows in yellow are for amino acids where preferred codons differ between the two sets of genes. Preferred codons identified in Sharp & Li 1987 (SL87) are listed in the last column for comparison [1]. The three amino acids where these differ from the top 25% set are in orange. Preferred codons remain the same across all columns for the remaining ten amino acids in blue.

Table S2. Z-scores of observed substitutions and chi-squared values for overall error rates per amino acid of the wild-type ribosomal variant

	R	H	K	D	E	S	T	N	Q	C	G	P	A	V	I	L	M	F	Y	W
R		-1	1	-2	-2.29	4.43	0.2	-0.96	-1.95	3	6.3	-2	0	-2	1	-1	-0	1	7.16	-0
H	-1		0	1	-4.39	-1.02	-2.97	0.44	-1.86	-1	-1	-3	-4	2	5	6	-1	-1	0.26	-1
K	-2	5		-3	4.29	5.77	-0.26	-3.7	9.37	-1	4.9	-3	-2	-2	-2	-3	3	-3	-1.31	1
D	-1	-1	-2		4.56		**24.47**	artifact	-4.67	11	-2	-4	-6	-6	1	1	2	-4	-1.53	-1
E	-2	-1	2	0		-0.53	artifact	-2.5	artifact	-0	-1	11	-4	-2	-4	-4	9	**15**	0.21	-1
S	-2	-2	-2	-6	artifact		5.33	-6.27	2.67	4	-1	-1	**36**	-7	-6	-7	-2	-4	-2.21	-0
T	-2	3	-2	2	-5.92	3.91		0.27	-3.19	-1	0	6	-5	**17**	-4	-5	-2	-2	-1.06	-1
N	-3	-1	-3	9	4.28	0.92	-5.97		4.93	-2	-1	5	-8	-6	1	-0	4	-1	-0.96	-1
Q	-1	-1	**16**	-4	10.71	1.36	-3.46	0.45		-2	-0	-4	-4	-2	-2	-2	2	-1	0.98	0
C	1	5	-0	-5	-4.1	-1.58	-4.72	-5.69	-0.41		-3	-3	-6	-3	9	**14**	-2	-3	8.71	0
G	-2	-2	-1	5	-7.47	2.17	8.83	5.21	-5.41	-0		-5	**21**	-3	-7	-8	-3	-5	-1.77	-1
P	2	3	-3	-3	-4.97	-1.6	-2.08	-0.12	-4.62	-2	-3		1	**20**	-3	-2	-3	-4	0.01	1
A	-2	-2	-3	-3	3.15	artifact	-3.34	1.83	4.12	-0	**16**	-2		2	-0	-3	1	-5	-2.09	-1
V	3	-2	-3	-4	-9.63	-1.71	-1.29	-4.08	-6.77	-3	-4	**14**	-8		**15**	**20**	-4	-6	-2.69	-1
X	5	-2	4	**11**	-2.1	-0.8	-5.3	4.98	7.38	-2	-3	-5	-6	7			-1	-3	-2.35	-1
M	-1	-0	-1	-2	**15.48**	-2.07	-5.74	**13.65**	-0.3	-2	-4	-6	-7	-6	-6	-7		8	0.9	0
F	1	-1	-1	-1	0.82	0.73	-2.21	1.16	6.61	0	-1	-2	-3	-2	-2	-2	6		artifact	**14**
Y	2	0	-1	-3	-2.02	-0.86	-1.62	-2.99	-1.38	1	0.3	-3	-3	-2	-1	-4	-1	**26**		1
W	2	-0	1	-1	-0.71	-0.41	-0.52	-1.56	-1.33	1	11	-1	-2	-1	-1	-2	-1	1	7.3	

Chi2	46	45	50	0	**199.1**	**263.2**	0.12	**288.9**	62.24	50	**318**	6	13	79	14	1	1	83	52.53	15

The upper matrix of values are the Z-scores determined by comparing the observed number of substitutions to the distribution of 10,000 simulated numbers of expected substitutions, dependent on marginal probabilities of sources and destinations. The top 5% of Z-scores are bolded. The lower matrix represents the chi-squared values of the translation error rates associated with each amino acid. The top 4 values are bolded.

Table S3. Z-scores of observed substitutions and chi-squared values for overall error rates per amino acid of the accurate ribosomal variant

	R	H	K	D	E	S	T	N	O	C	G	P	A	V	I	L	M	F	Y	W
R		0.9	6.8	-2	-2.01	3.81	-0.56	-1.63	-1.68	-1	11	-2	-1	-2	1.6	-1	-0	-1	4.38	-0
H	-1		-1	1.5	-4.73	-0.3	-2.63	-1.61	-2.3	-2	-1	-4	-5	2.2	7.4	7.6	-0	-2	-1.34	-1
K	-2	4.6		-2	4.03	4.36	-1.84	-3.3	10.94	-2	3.8	-2	-2	-0	-4	-3	3.5	-3	-1.25	-1
D	-2	-2	-2		8.27		**23.31**	artifact	-5.56	9.9	-2	-5	-6	-6	-0	0.6	2.9	-5	-1.6	-1
E	-2	-0	1.7	-2		0.32	artifact	-1.74	artifact	1.8	-2	9.6	-2	-0	-4	-4	5.8	**17**	0.62	1.9
S	-1	-2	-2	-6	artifact		8.39	-6.38	-1.01	1.6	-2	-1	**35**	-7	-7	-8	-3	-3	-2.2	0.6
T	0.7	4.1	-3	0.8	-5.95	0.51		1.75	-3.22	-0	-1	6.3	-6	**19**	-4	-4	-3	-1	0.63	-1
N	-2	-2	-3	13	3.33	0.41	-6.03		4.5	-3	-1	6.6	-8	-5	-0	-3	2.1	2.2	-0.64	-1
O	-2	-0	**14**	-4	12.2	1.57	-2.71	-1.08		-2	0.6	-4	-5	-1	-1	-3	0.8	0.6	2.61	0.1
C	1.6	5.3	-2	-4	-3.99	-1.04	-4.39	-5.54	-1.52		-1	-4	-6	-1	7.8	**16**	-2	-4	9.74	-1
G	-3	-2	-2	4.9	-8.08	2.42	0.61	6.68	-5.89	2.4		-5	**23**	-2	-7	-8	-1	-5	-1.44	-1
P	5	1	-3	-3	-4.38	-0.98	-1.18	0.13	-4.52	-2	-1		3.3	**16**	-1	-3	-3	-3	-1.87	-1
A	-2	-1	-1	-2	2.54	artifact	-2.21	2.89	3.81	-1	7.7	-2		4.8	-1	-2	0.4	-4	-1.84	-1
V	3.3	-2	-4	-6	-10.1	-2.5	-0.28	-3.29	-7.19	-3	-3	12	-10		**16**	**20**	-4	-6	-2.86	-1
X	2	-0	4.1	**14**	-5.53	-0.57	-4.43	4.97	**16.06**	-1	-2	-4	-5	-2			1.8	-4	-1.97	-1
M	-1	-1	0.8	-3	**16.78**	0.64	-5.46	**13.59**	2.07	-2	-2	-6	-8	-4	-7	-9		5.2	-0.02	0.3
F	-1	-1	-0	-2	1.77	-0.86	-1.78	2.11	3.29	-0	-1	-3	-3	0.7	-3	-3	9.4		artifact	**17**
Y	0.9	0.2	0.3	-3	-1.78	-0.95	-2.2	-3.44	-2.33	3.4	1.8	-3	-4	-2	-1	-3	-1	**29**		-1
W	4.1	-0	1.7	-2	-0.93	-0.47	-1.43	-1.74	-0.86	-1	**22**	-1	-2	-1	-1	-2	-1	-1	6.72	

Chi2	37	40	71	0	**149.2**	**250.8**	1.36	**363.6**	101.1	68	**398**	9	0.3	2.9	25	0.4	0.1	55	50.32	12

The upper matrix of values are the Z-scores determined by comparing the observed number of substitutions to the distribution of 10,000 simulated numbers of expected substitutions, dependent on marginal probabilities of sources and destinations. The top 5% of Z-scores are bolded. The lower matrix represents the chi-squared values of the translation error rates associated with each amino acid. The top 4 values are bolded.

Table S4. Z-scores of observed substitutions and chi-squared values for overall error rates per amino acid of the error-prone ribosomal variant

	R	H	K	D	E	S	T	N	O	C	G	P	A	V	I	L	M	F	Y	W
R		0.2	**12**	-1	-2.43	3.54	-1.42	-2.07	-0.49	-1	6.3	-1	-1	-2	1	-1	-1	-0	5.53	-0
H	2.3		0.3	-1	-4.55	-0.48	-2.36	-3.16	5.16	1.6	-1	-4	-5	2.5	5.6	3.2	-2	-0	0.61	0.6
K	-2	0.7		-5	1.41	2.11	-2.7	**19.53**	6.99	-1	0.7	-4	-4	-3	-4	-5	-1	-4	-1.84	1.2
D	-2	-3	-2		**12.28**		**23.61**	artifact	-4.45	6.3	6.2	-5	-6	-7	0.6	0.7	-0	-5	-1.15	-1
E	-3	-2	1.1	**33**		-2.05	artifact	-2.93	artifact	-1	-1	4.1	-7	-4	-7	-7	0.8	5.6	-1.48	-0
S	-2	-3	-2	-7	artifact		6.94	-7.29	1.77	1.8	-0	-1	**39**	-7	-7	-8	-3	-5	-2.49	-0
T	-1	0.7	-1	-1	-6.43	6.54		0.02	-3.75	-1	-2	4.6	-5	**23**	-5	-3	-3	-2	-1.32	-1
N	-2	-2	-2	7.6	1.26	1.89	-5.14		9.07	-2	-2	4.9	-8	-4	-1	-1	2.2	-1	-0.48	-1
O	0.4	**21**	11	-6	11.9	-0.69	-4.2	-2.19		-2	-1	-4	-6	-2	-3	-2	1.3	-1	0.38	0.9
C	4.2	2.7	-2	-6	-3.22	-1.09	-4.52	-6.02	0.53		-2	-3	-6	-2	10	**14**	-1	-4	8.52	-1
G	-1	-3	-2	1.1	-7.64	3.61	4.43	-1.82	-5.35	0.5		-4	**29**	-2	-6	-8	-2	-5	-1.82	-1
P	8.5	0.4	-2	-4	-4.09	-1.69	-0.27	0.04	-4.05	-2	-2		3.5	**16**	-1	-4	-2	-4	-0.42	-1
A	-2	-2	-2	-4	4.23	artifact	-2.36	4.01	-0.14	-0	9.5	-1		4.2	-1	-1	0.8	-5	-2.1	0.9
V	-0	-4	-4	-9	-10.5	-1.18	-0.55	-6.38	-6.88	-2	-4	**15**	-10		**20**	**25**	-5	-7	-3.07	-2
X	0.6	-3	0	4.4	-4.64	-1.14	-5.51	5.66	5.92	-2	-2	-4	-7	3			12	9.3	-2.43	-1
M	-2	-2	-0	-4	**16.49**	-2.4	-5.23	11.42	1.87	-1	-3	-6	-8	-3	-5	-9		5.6	1.73	0.1
F	-1	-1	0.9	-3	2.22	0.08	-1.93	4.95	1.53	-1	-1	-3	-3	-0	-2	-2	7.4		artifact	10
Y	-0	0.8	1.2	-4	-3.04	-1.05	-1.48	-3.5	-2.96	6.7	0.8	-3	-4	-3	-1	-2	-1	**29**		2.7
W	3.3	-1	0.2	-2	-1.75	3.19	-0.18	-1.96	-1.57	-0	5.1	-2	-1	0.8	-2	-1	-0	-1	**20.33**	
Chi2	89	2.8	87	94	**215.5**	**297.3**	2.42	**436.8**	48.45	0.8	**427**	3.2	1.5	4.8	11	3.5	2.1	146	56.99	16

The upper matrix of values are the Z-scores determined by comparing the observed number of substitutions to the distribution of 10,000 simulated numbers of expected substitutions, dependent on marginal probabilities of sources and destinations. The top 5% of Z-scores are bolded. The lower matrix represents the chi-squared values of the translation error rates associated with each amino acid. The top 4 values are bolded.

Table S5. Mistranslation events in the top 5% of Z-scores for the wild-type ribosomal variant

Amino Acid Source	Amino Acid Destination	Z-score	Minimum Distance (nt)
A	S	35.74	1
F	Y	26.29	1
T	D	24.47	2
A	G	21.33	1
V	P	20.23	2
L	V	19.63	1
V	T	16.50	2
G	A	16.11	1
K	Q	15.79	1
E	M	15.48	2
F	E	15.18	3
I	V	14.82	1
L	C	14.20	2
W	F	13.72	2
N	M	13.65	2
P	V	13.62	2
D	X	11.44	2
G	W	11.21	1

The significance (nominal P-value ≤ 0.001 and top 5% Z-score of observation) was determined by comparing the observed number of substitutions to the distribution of 10,000 simulated values of expected substitutions, dependent on the marginal probabilities of sources and destinations. The distance was defined by the minimum number of nucleotides per codon required to be misread to cause the observed translation error.

Table S6. Mistranslation events in the top 5% of Z-scores for the accurate ribosomal variant

Amino Acid Source	Amino Acid Destination	Z-score	Minimum Distance (nt)
A	S	35.14	1
F	Y	29.47	1
T	D	23.31	2
A	G	23.07	1
G	W	22.10	1
L	V	20.17	1
V	T	18.84	2
W	F	17.22	2
F	E	17.14	3
E	M	16.78	2
V	P	16.39	2
I	V	16.21	1
L	C	16.08	2
Q	X	16.06	1
D	X	14.04	2
N	M	13.59	2
K	Q	13.55	1
D	N	12.92	1

The significance (nominal *P*-value $\leq$0.001 and top 5% Z-score of observation) was determined by comparing the observed number of substitutions to the distribution of 10,000 simulated values of expected substitutions, dependent on the marginal probabilities of sources and destinations. The distance was defined by the minimum number of nucleotides per codon required to be misread to cause the observed translation error.

Table S7. Mistranslation events in the top 5% of Z-scores for the error-prone ribosomal variant

Amino Acid Source	Amino Acid Destination	Z-score	Minimum Distance (nt)
A	S	38.74	1
D	E	32.59	1
F	Y	29.17	1
A	G	29.13	1
L	V	24.64	1
T	D	23.61	2
V	T	22.80	2
H	Q	21.02	1
Y	W	20.33	2
I	V	19.69	1
N	K	19.53	1
E	M	16.49	2
V	P	15.56	2
P	V	15.37	2
L	C	13.64	2
E	D	12.28	1
K	R	12.03	1
E	Q	11.90	1

The significance (nominal P-value < or = 0.0001 and top 5% Z-score of observation) was determined by comparing the observed number of substitutions to the distribution of 10,000 simulated numbers of expected substitutions, dependent on marginal probabilities of sources and destinations. The distance was defined by the minimum number of nucleotides required to be misread to cause the translation error observed.

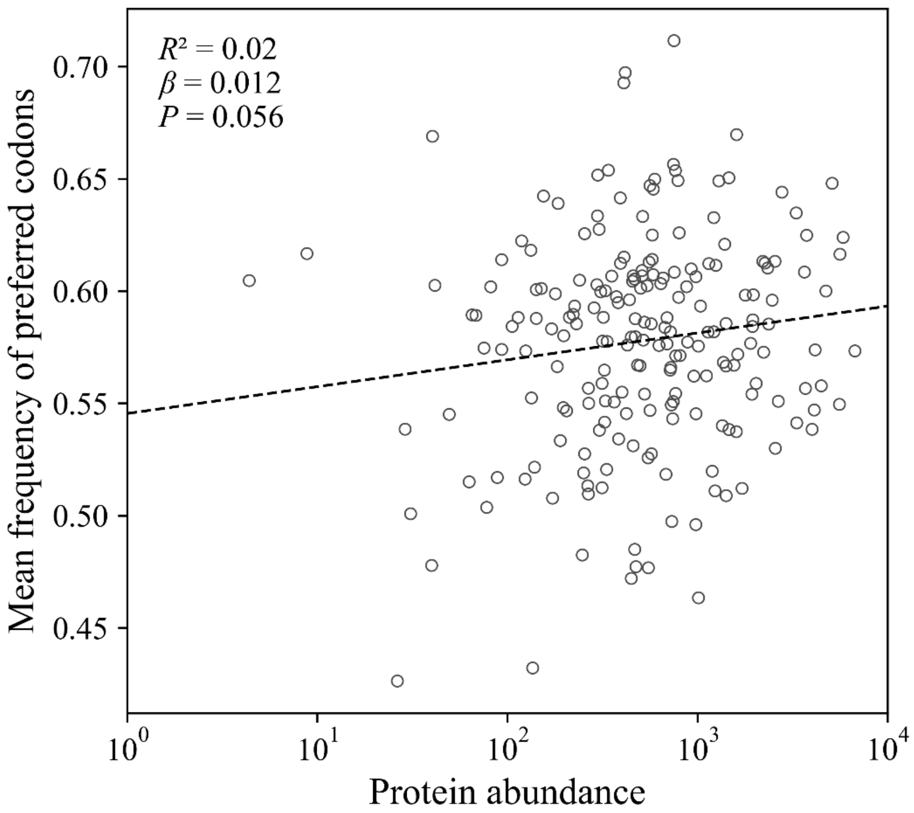

Figure S1. The mean frequency of preferred codons among synonymous codons in a gene are uncorrelated with its protein abundance, for the set of preferred codons predicted based on codon frequencies of all genes (PC2). The linear regression coefficient for a correlation between this measure of codon usage bias and log protein abundance for 205 genes (data from PaxDb [3]) was $\beta = 0.012$ (SE = 0.006, $R^2 = 0.02$, $P = 0.056$) and the Spearman rank correlation coefficient was $\rho = 0.102$ ($P = 0.15$). This figure is comparable to main Figure 3A which shows an enrichment of preferred codons with increasing protein abundance, for the set of preferred codons predicted based on codon frequencies of highly expressed genes

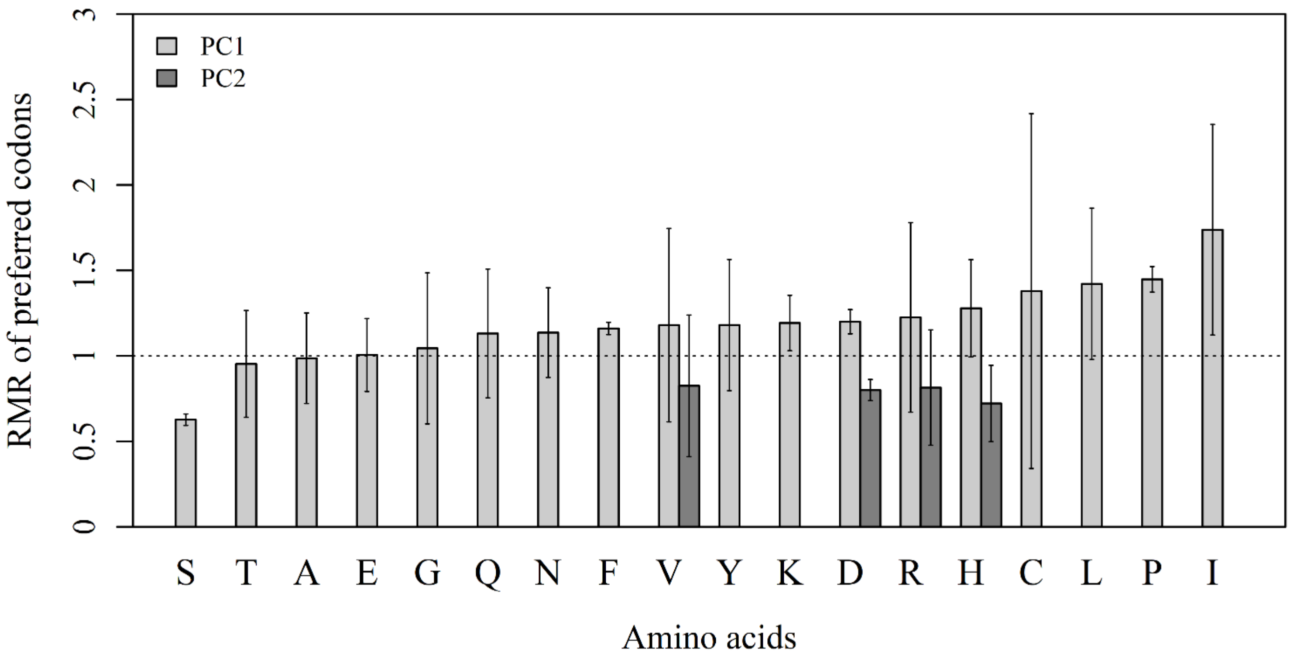

Figure S2. RMR of preferred codons of 18 amino acids, for the two sets of preferred codons - predicted based on codon frequencies of highly expressed genes (PC1), or of all genes (PC2). Preferred codons, that differ between these two sets for four amino acids, have lower mean RMR in PC2. The error bars denote one standard error of the mean RMR over three biological replicates.

A

Source Amino Acid (columns); Destination Amino Acid (rows)

	R	H	K	D	E	S	T	N	Q	C	G	P	A	V	I	L	M	F	Y	W
R		3.03	6.79	0.00	0.28	7.42	0.73	0.34	0.18	0.00	13.00	0.28	0.64	0.28	2.23	1.10	0.72	0.70	6.65	0.00
H	2.38		1.11	4.19	0.00	3.70	0.72	1.93	1.27	0.00	1.39	0.00	0.00	4.08	11.06	10.33	2.54	1.03	0.00	0.00
K	0.00	14.99		0.97	5.18	16.79	1.21	0.53	12.55	0.00	9.05	0.93	1.31	2.62	0.28	1.14	7.11	0.00	0.00	0.00
D	3.74	0.00	3.57		14.72		38.19		0.19	30.50	0.00	1.34	1.21	0.69	7.95	8.14	13.44	0.00	2.08	0.00
E	0.00	1.39	7.30	1.72		3.92		2.27		5.27	0.00	11.18	2.21	3.41	0.69	0.64	12.37	22.48	5.62	14.44
S	4.24	0.00	2.46	0.00			20.25	0.74	6.85	9.55	1.85	7.55	39.17	0.41	0.00	0.11	1.97	2.78	0.00	6.67
T	5.89	24.42	0.00	5.33	0.13	5.68		6.44	2.37	3.31	2.67	12.78	0.34	24.49	1.52	1.64	0.00	3.07	7.70	0.00
N	4.68	1.39	1.35	30.78	15.09	12.68	1.45		16.77	5.56	10.22	20.57	1.01	3.65	10.59	9.08	16.10	15.97	7.68	0.00
Q	2.55	2.78	33.29	1.01	16.20	9.39	2.43	4.69		0.00	7.19	0.48	1.38	4.38	5.57	3.98	7.35	6.55	12.88	3.33
C	8.31	21.93	1.59	1.32	2.59	3.70	0.74	0.53	5.49		2.31	1.61	0.33	5.23	11.61	17.16	1.30	0.66	38.08	0.00
G	0.61	0.00	5.75	15.45	0.26	18.77	9.92	16.21	0.74	21.64		1.72	39.98	6.99	0.42	0.11	6.71	0.00	2.90	0.00
P	18.31	10.57	0.00	2.27	1.82	3.70	4.38	5.86	0.57	0.78	2.31		9.07	22.45	4.32	3.49	0.00	1.06	0.00	0.00
A	0.76	4.76	3.06	3.41	7.93		3.17	8.56	8.35	2.74	24.00	3.19		10.34	4.86	4.17	6.21	0.00	0.00	0.00
V	20.91	4.42	0.79	3.05	0.00	0.00	13.14	8.38	1.46	0.00	1.28	36.46	0.54		35.82	37.47	0.67	0.00	0.00	0.00
X	11.43	2.78	17.54	22.70	1.34	3.51	0.98	11.57	23.15	3.92	0.00	0.76	1.55	3.99			10.35	0.64	0.00	0.00
M	7.51	2.78	10.44	7.02	30.73	10.73	1.71	28.98	15.43	1.55	2.67	0.96	1.17	4.22	1.55	0.43		19.81	10.40	14.44
F	1.19	0.00	1.35	0.78	2.37	0.00	0.49	2.96	3.71	5.56	0.00	0.00	0.00	1.66	0.28	0.59	11.72			61.11
Y	3.74	4.76	1.67	0.00	1.06	0.00	0.49	0.00	0.56	9.64	4.06	0.00	0.10	0.97	1.11	0.22	1.45	25.24		0.00
W	3.74	0.00	1.94	0.00	0.30	0.00	0.00	0.00	0.36	0.00	17.98	0.19	0.00	0.14	0.15	0.20	0.00	0.00	6.01	

B

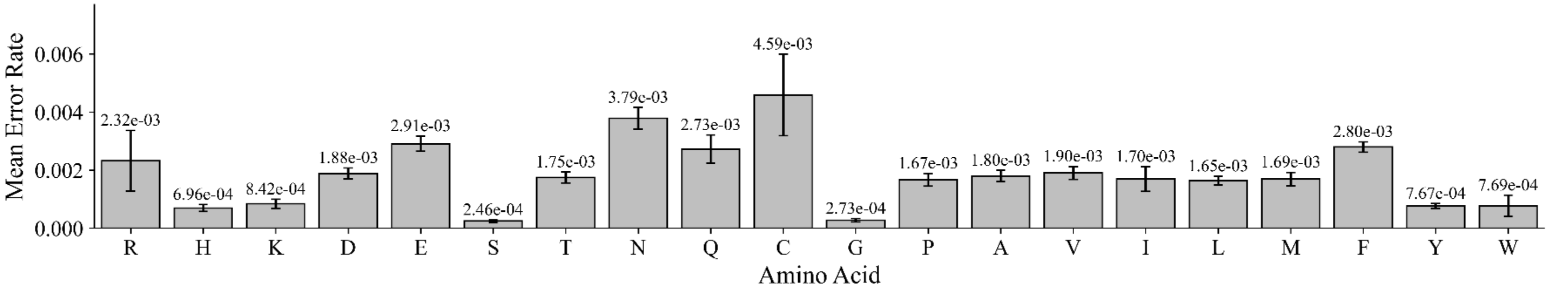

Figure S3. Mean rate and spectrum of translation errors in the accurate ribosomal variant of *E. coli*. A) Wild-type (source) amino acids are represented as columns, and erroneously incorporated (destination) amino acids are represented as rows. B) The mean error rate at which each amino acid is mistranslated is plotted with standard error bars across three biological replicates. Substitutions that are indistinguishable from chemical modifications of the source amino acid (see Table 3) are removed from the analysis (grey boxes). Leucine and isoleucine are combined in the destination row "X." Subfigure B gives the average rate at which each amino acid is mistranslated, while subfigure A gives the percentage distribution of those mistranslations across destination amino acids.

A

Source Amino Acid (columns); Destination Amino Acid (rows)

Destination \ Source	R	H	K	D	E	S	T	N	Q	C	G	P	A	V	I	L	M	F	Y	W
R		0.81	9.48	0.42	0.13	5.38	0.27	0.14	0.63	0.00	5.90	0.25	0.58	0.11	1.49	0.68	0.00	0.74	6.11	0.00
H	6.90		3.36	2.09	0.31	1.75	0.90	0.86	7.75	8.33	1.36	0.00	0.00	4.13	8.23	6.03	0.54	2.68	5.00	11.11
K	1.04	6.60		0.95	5.82	11.26	1.89	20.38	12.00	0.00	5.78	0.43	1.86	1.80	1.38	1.01	3.01	0.00	0.00	6.06
D	1.08	0.00	4.15		17.49		38.76		1.71	32.09	21.80	1.36	1.71	0.54	9.51	9.00	6.73	0.23	5.00	0.00
E	0.00	0.81	10.69	38.05		0.00		4.49		5.15	5.66	10.97	0.74	3.17	0.57	1.29	9.06	15.16	2.78	3.03
S	3.92	0.00	4.15	1.52			17.63	0.41	9.88	11.47	7.77	7.89	40.33	0.47	0.57	0.45	1.49	1.17	0.00	11.11
T	3.16	5.66	1.96	3.89	0.00	23.40		4.58	1.01	1.45	1.36	9.60	0.93	25.47	0.92	2.98	0.54	2.31	1.11	0.00
N	3.46	3.64	6.23	19.20	11.05	17.87	2.08		21.24	2.78	6.02	15.97	0.69	5.14	9.12	9.02	14.34	8.78	7.78	0.00
Q	6.61	55.93	27.50	1.08	15.34	3.33	0.97	3.76		0.00	4.43	0.85	0.41	4.22	3.56	5.36	7.96	4.99	6.39	9.70
C	13.46	10.41	0.79	0.44	2.92	1.96	0.23	0.00	7.19		0.62	2.21	0.17	3.50	12.31	14.12	3.48	0.95	27.78	0.00
G	3.17	0.00	3.97	7.85	0.34	18.98	12.65	5.46	0.47	8.05		2.37	39.41	5.07	0.61	0.24	2.32	0.46	1.11	0.00
P	24.33	6.08	1.77	1.87	2.00	0.00	4.98	5.11	0.86	0.00	0.74		8.71	16.95	4.15	1.87	1.39	1.20	3.61	0.00
A	1.04	0.00	0.98	2.06	9.38		3.28	9.59	5.74	5.15	26.67	4.67		9.96	4.88	5.08	7.08	0.00	0.00	9.70
V	9.52	0.00	1.96	1.93	0.49	7.05	11.89	4.21	1.33	0.00	0.00	39.62	0.39		36.81	40.11	1.44	0.27	0.00	0.00
X	10.62	0.00	7.10	12.15	3.14	3.92	0.45	13.64	14.05	0.00	2.94	3.39	1.65	11.31			30.51	20.65	0.00	0.00
M	5.28	6.20	10.69	6.28	28.28	0.00	2.09	23.22	13.25	4.23	1.60	0.43	1.86	5.82	4.41	0.47		18.94	15.56	9.70
F	1.08	0.00	1.96	0.21	2.63	1.67	0.48	4.02	2.88	0.00	0.85	0.00	0.17	1.30	0.52	1.28	8.59			33.54
Y	2.15	3.85	2.75	0.00	0.54	0.00	1.01	0.14	0.00	21.30	2.71	0.00	0.00	0.28	0.94	0.62	1.08	21.24		6.06
W	3.19	0.00	0.49	0.00	0.13	3.42	0.45	0.00	0.00	0.00	3.80	0.00	0.39	0.77	0.00	0.39	0.44	0.23	17.78	

B

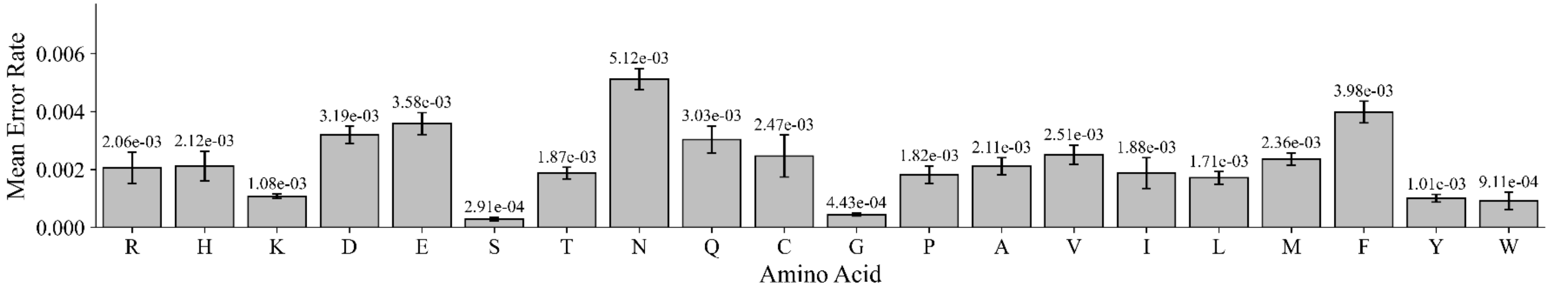

Figure S4. Mean rate and spectrum of translation errors in the error-prone ribosomal variant of *E. coli*. A) Wild-type (source) amino acids are represented as columns, and erroneously incorporated (destination) amino acids are represented as rows. B) The mean error rate at which each amino acid is mistranslated is plotted with standard error bars across three biological replicates. Substitutions that are indistinguishable from chemical modifications of the source amino acid (see Table 3) are removed from the analysis (grey boxes). Leucine and isoleucine are combined in the destination row "X." Subfigure B gives the average rate at which each amino acid is mistranslated, while subfigure A gives the percentage distribution of those mistranslations across destination amino acids.

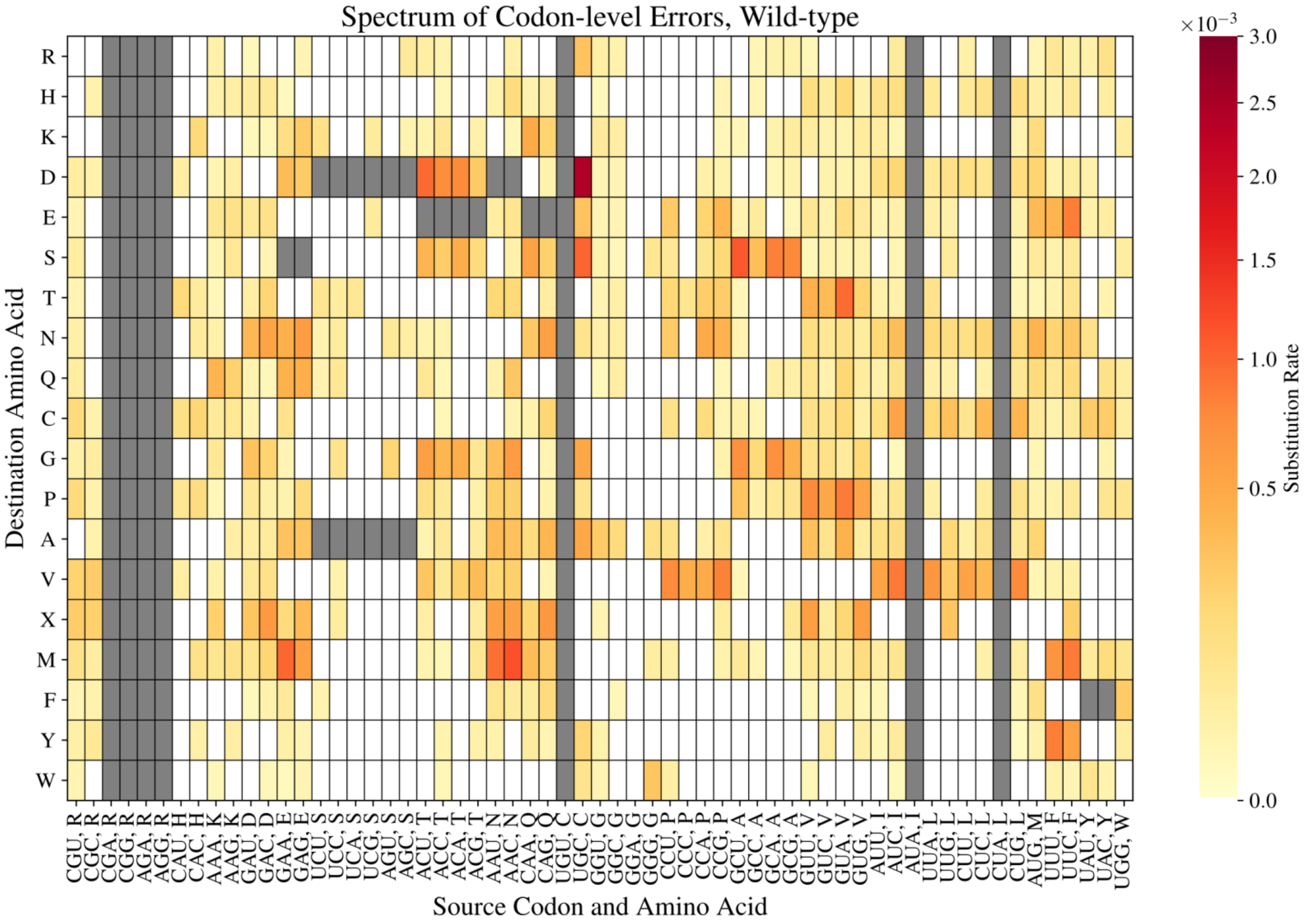

Figure S5. Mean codon-specific substitution rate of the wild-type ribosomal variant of *E. coli*. Codon results are filtered to include only those with at least 3,000 sampled codons. Grey boxes denote that the result was filtered due to either chemical artifact (see Table 3), or failure to meet the sampling cutoff. Substitution bias, used in the calculation for substitution rate, is the fraction of observed errors from a source codon that are directed to a specific destination amino acid. The codon-specific substitution rate is calculated as the product of the mean codon error rate and the mean substitution bias of a source codon toward each possible destination amino acid.

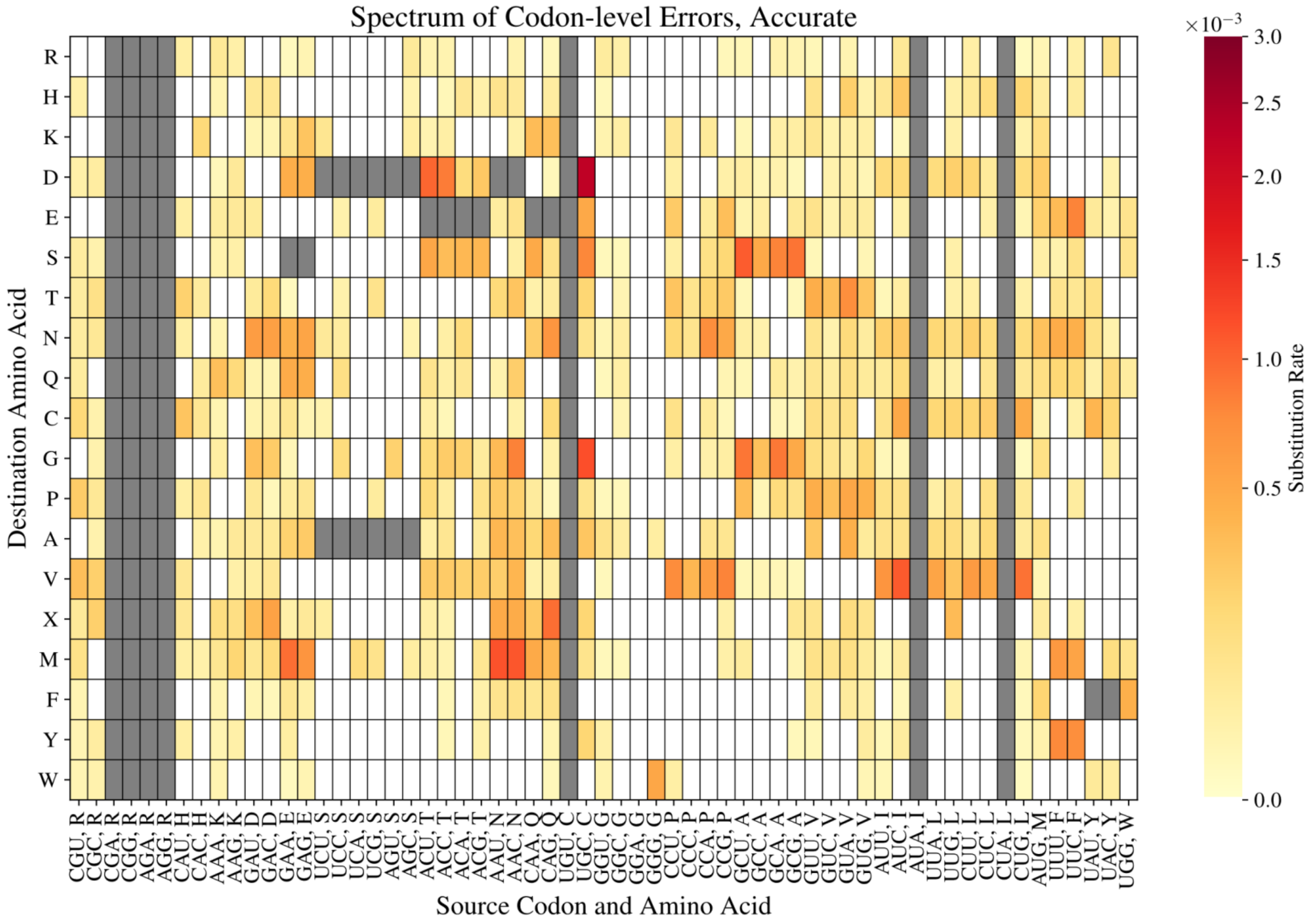

Figure S6. Mean codon-specific substitution rate of the accurate ribosomal variant of *E. coli*. Codon results are filtered to include only those with at least 3,000 sampled codons. Grey boxes denote that the result was filtered due to either chemical artifact (see Table 3), or failure to meet the sampling cutoff. Substitution bias, used in the calculation for substitution rate, is the fraction of observed errors from a source codon that are directed to a specific destination amino acid. The codon-specific substitution rate is calculated as the product of the mean codon error rate and the mean substitution bias of a source codon toward each possible destination amino acid.

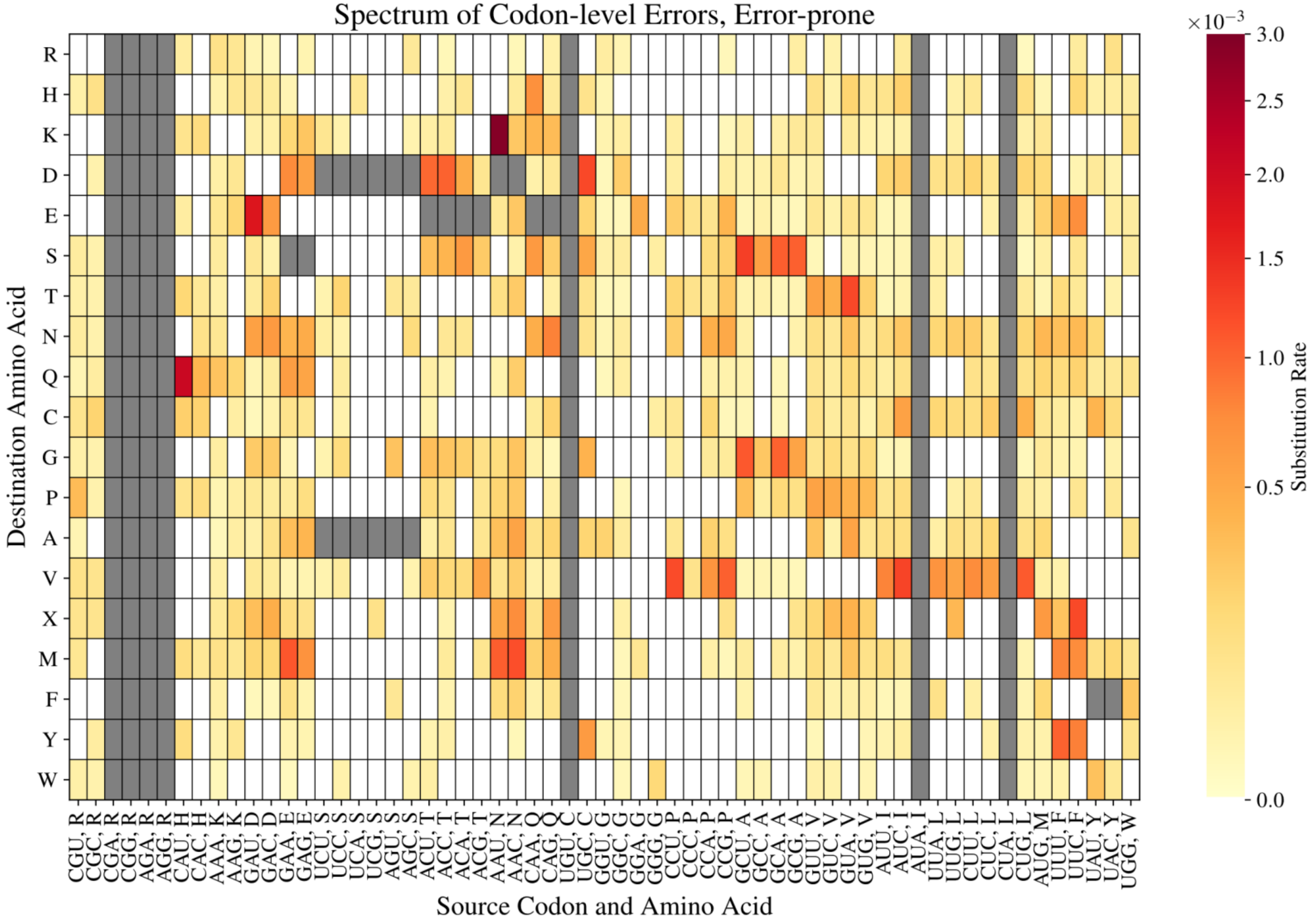

Figure S7. Mean codon-specific substitution rate of the error-prone ribosomal variant of *E. coli*. Codon results are filtered to include only those with at least 3,000 sampled codons. Grey boxes denote that the result was filtered due to either chemical artifact (see Table 3), or failure to meet the sampling cutoff. Substitution bias, used in the calculation for substitution rate, is the fraction of observed errors from a source codon that are directed to a specific destination amino acid. The codon-specific substitution rate is calculated as the product of the mean codon error rate and the mean substitution bias of a source codon toward each possible destination amino acid.

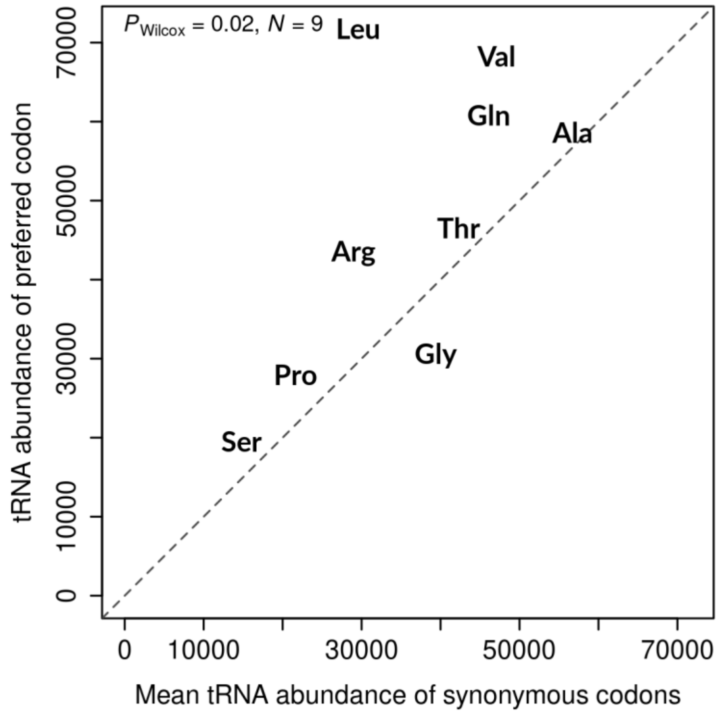

Figure S8. Preferred codons correspond to more abundant tRNA species. The tRNA abundance of the preferred codon for each amino acid is greater than the mean tRNA abundance over its synonymous codons for all amino acids except Gly. This analysis was done for nine amino acids with available tRNA abundance data [4] for more than one iso-accepting tRNA species, which were identified on the basis of the revised wobble rules for the four bases – A,C,G, and U [5].

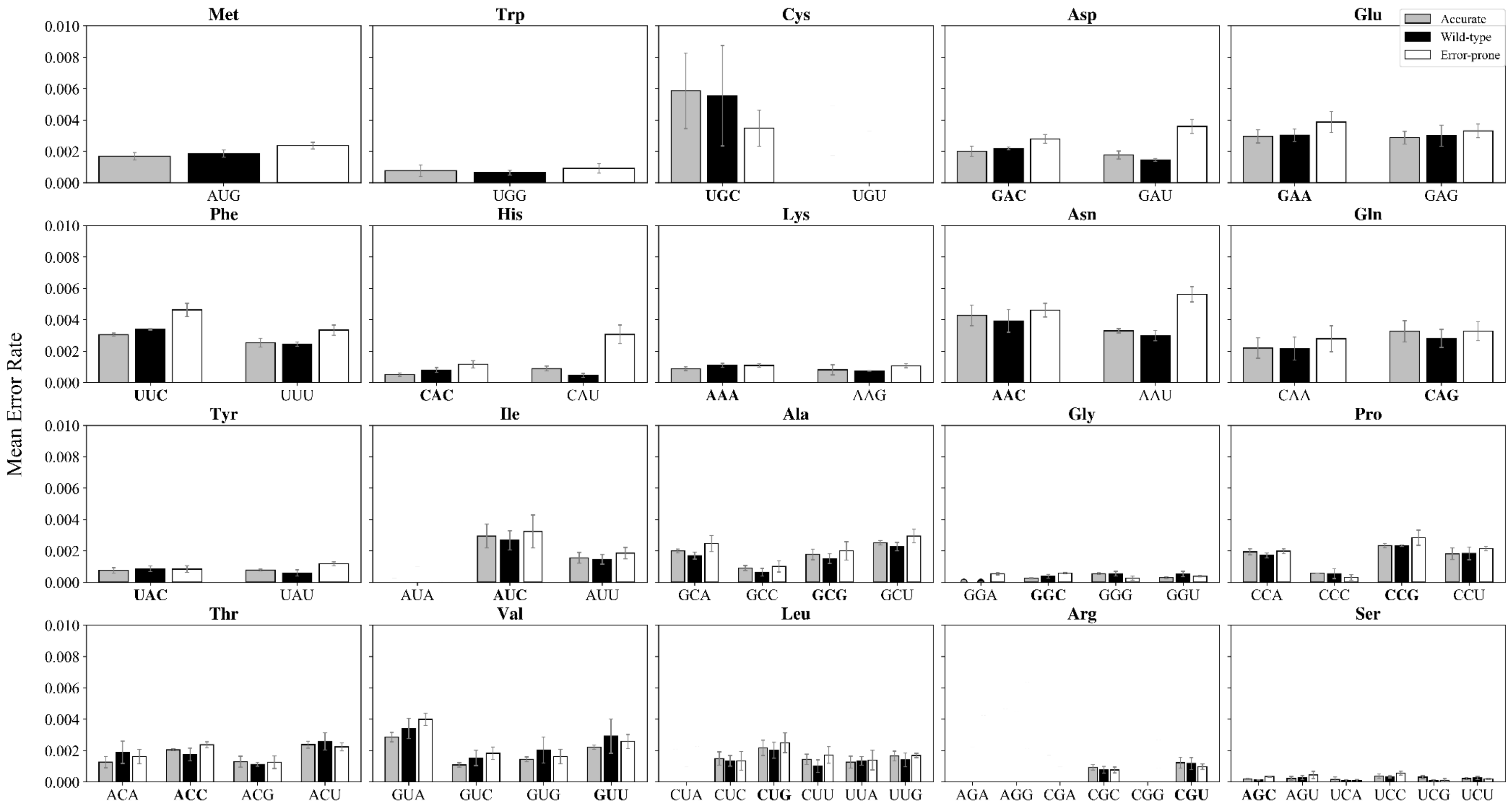

Figure S9. Mean error rates of each codon according to the ribosomal variant of *E. coli*. A minimum of 3,000 sampled codons is required per each plotted result. Mean error rates are plotted here with standard error bars across three biological replicates. Codons with measured error rates of zero are plotted as points (e.g. GGA). Preferred codons are indicated in bold. Ribosomal variants are distinguished by the colors shown in the legend.

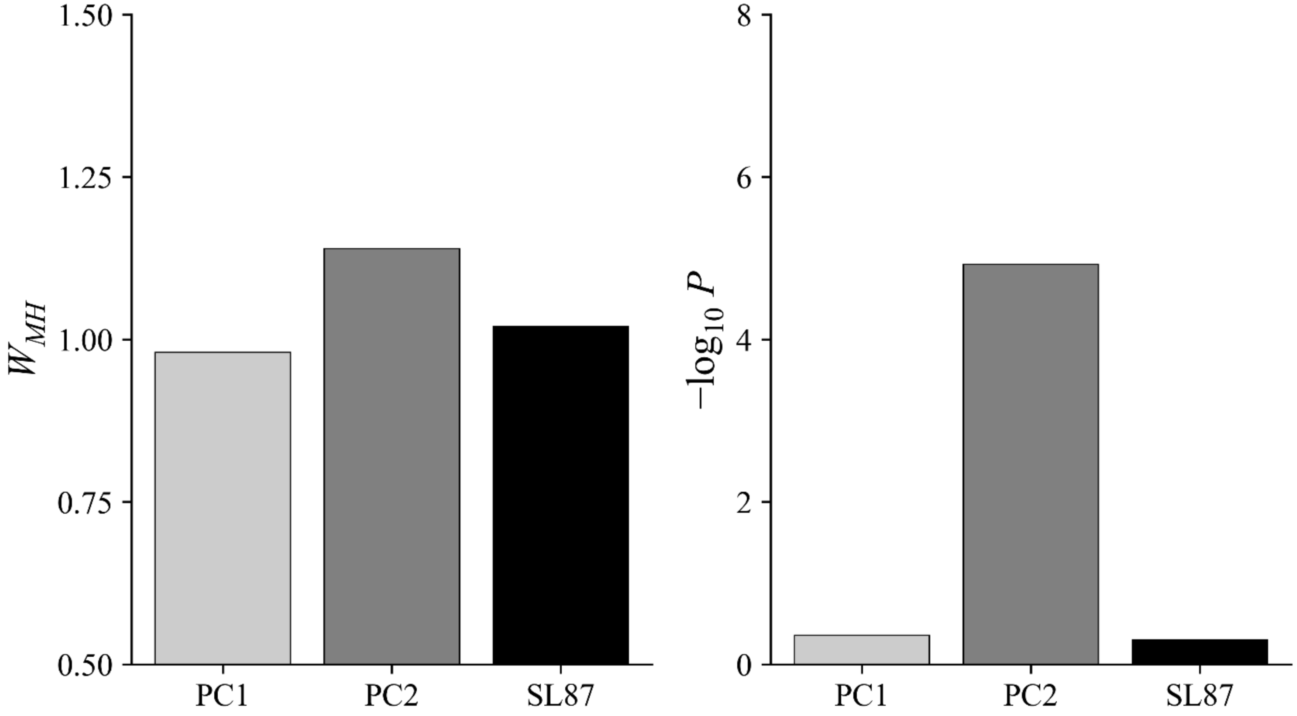

Figure S10. Akashi's test of enrichment of preferred codons at conserved amino-acid sites [6] for three sets of preferred codons. PC1 and PC2 are predicted based on codon frequencies of highly expressed genes and of all genes, respectively. SL87 is the set of preferred codons predicted by Sharp and Li (1987). W_{MH} is the Mantel-Haenszel's statistic for the enrichment of preferred codons at conserved sites relative to variable sites (Methods) [7]. This is equivalent to an Odds Ratio (OR) for the counts of preferred and non-preferred synonymous codons pooled across different amino acids and genes. $W_{MH} > 1$ indicates an enrichment of preferred codons at conserved sites. Log-transformed *P*-values are shown to the right, where a value greater than 2 indicates a significant result ($P < 0.01$); PC2 reaches significance ($P = 1.2 \times 10^{-5}$).

References

1. Sharp PM, Li W-H. The rate of synonymous substitution in enterobacterial genes is inversely related to codon usage bias. *Mol. Biol. Evol.* 1987;4:222–230. https://doi.org/10.1093/oxfordjournals.molbev.a040443.

2. Bulmer M. Coevolution of codon usage and transfer RNA abundance. *Nature* 1987;325:728–730. https://doi.org/10.1038/325728a0.

3. Wang M, Weiss M, Simonovic M, *et al.* PaxDb, a database of protein abundance averages across all three domains of life. *Mol. Cell. Proteomics* 2012;11:492–500. https://doi.org/10.1074/mcp.O111.014704.

4. Wei Y, Silke JR, Xia X. An improved estimation of tRNA expression to better elucidate the coevolution between tRNA abundance and codon usage in bacteria. *Sci. Rep.* 2019;9:3184. https://doi.org/10.1038/s41598-019-39369-x.

5. Murphy FV, Ramakrishnan V. Structure of a purine-purine wobble base pair in the decoding center of the ribosome. *Nat. Struct. Mol. Biol. 2004 1112* 2004;11:1251–1252. https://doi.org/10.1038/nsmb866.

6. Akashi H. Synonymous codon usage in *Drosophila melanogaster*: natural selection and translational accuracy. *Genetics* 1994;136:927–935. https://doi.org/10.1093/genetics/136.3.927.

7. Mantel N, Haenszel W. Statistical aspects of the analysis of data from retrospective studies of disease. *J. Natl. Cancer Inst.* 1959;22:719–48. https://doi.org/doi.org/10.1093/jnci/22.4.719.
